# A moral house divided: How idealized family models impact political cognition

**DOI:** 10.1371/journal.pone.0193347

**Published:** 2018-04-11

**Authors:** Matthew Feinberg, Elisabeth Wehling

**Affiliations:** 1 Rotman School of Management, University of Toronto, Toronto, ON, Canada; 2 University of California, Berkeley, CA, United States of America; Saint Peter's University, UNITED STATES

## Abstract

People’s political attitudes tend to fall into two groups: progressive and conservative. Moral Politics Theory asserts that this ideological divide is the product of two contrasting moral worldviews, which are conceptually anchored in individuals’ cognitive models about ideal parenting and family life. These models, here labeled the strict and nurturant models, serve as conceptual templates for how society should function, and dictate whether one will endorse more conservative or progressive positions. According to Moral Politics Theory, individuals map their parenting ideals onto the societal domain by engaging the nation-as-family metaphor, which facilitates reasoning about the abstract social world (the nation) in terms of more concrete world experience (family life). In the present research, we conduct an empirical examination of these core assertions of Moral Politics Theory. In Studies 1–3, we experimentally test whether family ideals directly map onto political attitudes while ruling out alternative explanations. In Studies 4–5, we use both correlational and experimental methods to examine the nation-as-family metaphor’s role in facilitating the translation of family beliefs into societal beliefs and, ultimately, political attitudes. Overall, we found consistent support for Moral Politics Theory’s assertions that family ideals directly impact political judgment, and that the nation-as-family metaphor serves a mediating role in this phenomenon.

## Introduction

Even though societies face an assortment of social and economic challenges that often appear unrelated, individuals’ attitudes on how exactly a nation should proceed in tackling these diverse challenges tend to fit into one of two major camps: conservative “right-leaning” or progressive “left-leaning”. Yet, as widespread as this division tends to be, where conservative and progressive attitudes come from has been a puzzle, and explaining the underlying roots of this ideological division has become an important topic in the cognitive and social sciences.

**Fig 1:**
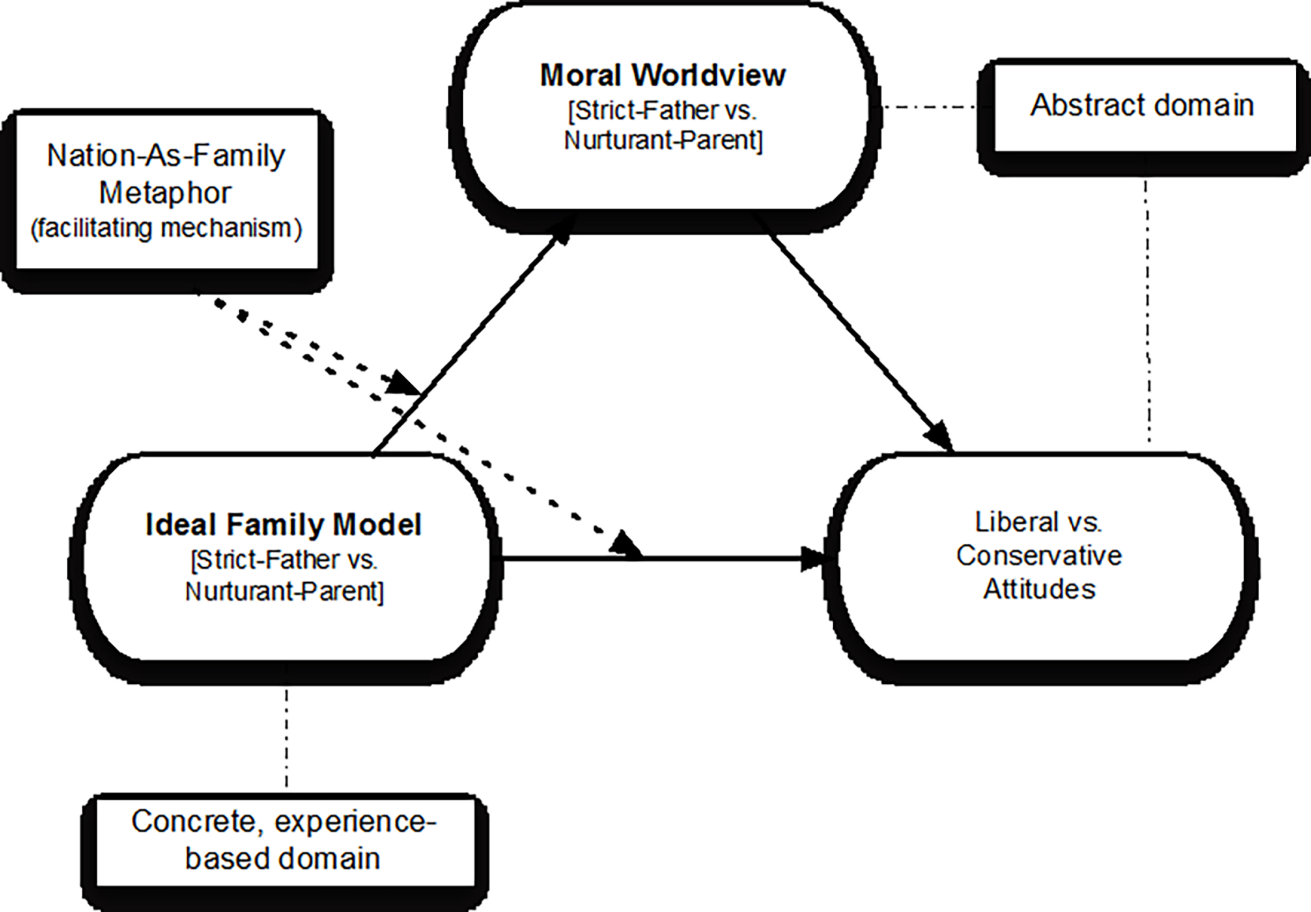
Moral Politics Theory model showing how individuals’ ideal family models, strict or nurturant, translate into moral worldviews, which in turn guide political attitudes.

One early account of the foundations of conservative and progressive attitudes is Moral Politics Theory (MPT, henceforth) [1]. MPT holds that conservatives and progressives possess different moral worldviews, which conceptually unify their positions on issues as diverse as abortion, education, the economy, and the environment. These worldviews stem from different idealized family models referred to as the “strict-father” and “nurturant-parent” models (we label them the “strict model” and “nurturant model” henceforth. See S1 File for a discussion on the gendered nature of these models). These models encompass notions about the nature of children, ideal traits in children, and ideal parenting, and they serve as a conceptual template for political reasoning, with endorsement of the strict model resulting in more conservative attitudes and endorsement of the nurturant model resulting in more progressive attitudes. At the core of MPT lies the assertion that people inherently rely on their notions of ideal family life when reasoning about how society should function, which, in turn, informs their political attitudes. The mechanism underlying this phenomenon, MPT argues, is the use of a conceptual metaphor whereby individuals tend to construe nationhood in terms of a family (see Fig 1).

Politicians and their strategists have commonly turned to MPT as a guide for political maneuvering, and the theory has influenced numerous international political campaigns (e.g., [2]). As Speaker of the House, Nancy Pelosi declared that “Lakoff’s ideas ‘forever changed’ the way in which Democratic House members reason about politics” [3]. However, as popular as MPT has become in the political world, many of its key predictions still lack comprehensive empirical foundation [4–7]. Recent investigations have found empirical support that the strict and nurturant models are coherent and distinct belief systems, which correlate with conservative and progressive attitudes, respectively [8, 9] (also see [4, 7, 10–15]). However, research has yet to explore MPT’s fundamental assertions that family-level beliefs causally influence nation-level political attitudes, and that the nation-as-family metaphor largely drives this causal link–the focus of the present investigation.

### Moral Politics Theory

Recent research in the cognitive and social sciences has established that moral beliefs are closely associated with one’s political thinking and behavior (e.g., [16–19]). For instance, moral concerns help explain political engagement [20, 21], policy and candidate evaluation [22], susceptibility to political influence [23–25], and progressive or conservative attitudes [26, 27]. Although such research has greatly advanced our understanding of the role individual differences in morality play for political judgment, it has not addressed why concerns with certain moral values, but not others, should co-occur with one another to form larger ideological constructs, and what the nature of such interconnected values at the foundation of conservatism and progressivism would be.

MPT [1] not only suggests a unique set of values at the basis of conservatism and progressivism, it also helps account for the coalescence of conservative and progressive moral beliefs into larger, coherent models of morality and lends insight into their cognitive foundations and experiential basis. The theory builds on the finding that the mind primarily processes abstract concepts in terms of knowledge acquired through concrete interaction with the world (e.g., [28–32]). Specifically, in line with Conceptual Metaphor Theory [33], MPT proposes that people’s beliefs about ideal family life serve as a conceptual anchor for their larger, moral belief systems about how society and its members should function, and these moral belief systems dictate people’s political attitudes. The theory holds that the translation of family-level beliefs into nation-level attitudes occurs via the nation-as-family metaphor. This metaphor facilitates reasoning about the highly abstract world of society and politics (the metaphoric target domain) by linking it to the more concrete, experience-based knowledge individuals have about how families function (the metaphoric source domain). Through the nation-as-family metaphor, people’s beliefs about ideal traits in children translate into beliefs about ideal traits in citizens. Likewise, their beliefs about ideal parenting translate into beliefs about ideal governance (see Fig 1) [1, 34].

### Two worldviews as organizing principles for moral beliefs

MPT proposes that conservative and progressive attitudes are grounded in idealized cognitive models that serve as “moral worldviews”, i.e., as organizing principles for larger sets of moral concerns. Lakoff [1] labels these moral worldviews the strict and nurturant models.

#### The strict model

The strict model [1] builds on the belief that the world is a dangerous and competitive place. It revolves around strictness and emphasizes withstanding temptation, abiding to authorities, and controlling oneself for the sake of maximal self-discipline and independence. Children are taken to have a natural tendency toward misbehavior and self-indulgence, and it is the parent’s obligation to teach them self-reliance, self-discipline, and obedience. This is best accomplished through strict rule-enforcement, harsh punishment, and absolute parental authority. Furthermore, communication and decision-making within the family should be maximally hierarchical, and children should never question the authority of their parents. Behavioral standards set forth by the parents are seen as absolute rights and wrongs. Competition is seen as inherently moral, as it leads to self-discipline. Giving children things that they have not earned is bad parenting, because it makes them weak and dependent. It is assumed that events or outcomes in the world have direct, simple causes, so that, for example, a lack of success in children is a direct result of a lack of their self-discipline. According to the strict model, loving and nurturing one’s children is crucial, but in the case of misbehavior or lack of success priority must be given to punishment. This “tough love” is seen as an expression of concern for the child, and parents who unconditionally nurture and love their children are seen as immoral, as they impair their children’s growth. Further, authority is gendered. Since men are seen as being natural authorities and being more capable of implementing punishment than women, they are typically the primary authority in the family. Finally, once children have become their own moral authorities, parents should no longer meddle with their lives, as it is considered immoral to interfere with adults who have successfully become their own “strict-endorsers”.

#### The nurturant model

The nurturant model [1] builds on the belief that children are born good and that their good nature must be fostered. It revolves around nurturance and emphasizes empathy, care for oneself and others, acknowledgment of individual needs and viewpoints, and personal happiness and self-fulfillment. Empathy, nurturance, and cooperation are seen as important moral traits, and in order to foster them, parents must lead by example and be nurturers who empathize with their children and understand their individual needs. Children must learn to take care of themselves and others. Self-responsibility is seen as the basis for social responsibility. The model holds that children need different types of support from their parents, and since the ultimate goal of parenting is to nurture children and empower them to follow their dreams, children must be given what they need, not what they presumably “deserve”. Through being recognized as individuals, children learn to empathize with others. Furthermore, parental decisions and rules should be discussed with children in open, respectful, two-way communication, and children’s obedience to parental authority should come out of love and respect, not the fear of punishment. This parenting is seen as fostering important traits in children, such as openness, accountability, and respect for others. Moreover, it is assumed that events or behaviors, such as a child struggling in life, often have systemic causes that are highly complex. Tolerance is an important moral trait, and parents have to teach their children to see the world through other people’s eyes. Being able to successfully connect and cooperate with others and knowing to never inflict harm are seen as inherently moral traits. A principal way of teaching children these traits is to lead by example and never psychologically or physically harm one’s children. Finally, the model calls for a life-long, cooperative, and mutually caring relationship between children and parents.

### Mapping the models onto the social domain

MPT holds that these family models map onto individuals’ moral reasoning about the social world more generally, and that the endorsement of the strict and nurturant models therefore map onto, and form the basis of, conservative and progressive values, respectively. For instance, individuals who ascribe to the strict parenting model will ascribe to a strict worldview and will likely have strong moral intuitions about policies that, in their eyes, constitute “immoral indulgence” of the citizenry and compromise the values of “self-reliance” and “self-discipline”, such as public healthcare, public education, welfare, or even food stamps. Likewise, individuals who ascribe to the nurturant parenting model will ascribe to a nurturant worldview and will likely have strong moral intuitions about policies that, in their eyes, violate moral concerns with “empathy,” “need-based fairness,” and “empowerment”, and thus more strongly support social welfare, public healthcare, and public education as means to act on one’s empathy and empower citizens to live fulfilled lives.

Since MPT was first introduced [1], research has empirically established the strict and nurturant models as unified belief systems that are conceptually independent of each other [9]. Moreover, studies found support for MPT’s assertion that these two models predict conservative and progressive political attitudes. For instance, studies using linguistic analyses to infer strict and nurturant ideals have found a clear link between the family models and political ideology [4, 7, 8, 11, 12, 14, 15]. Additionally, a study measuring a handful of the models’ components found a correlation between strict and nurturant family beliefs and political attitudes [10], and, most recently, a comprehensive study examining the two models in their entirety confirmed the robust association between the models and political attitudes [9].

#### The nation-as-family metaphor

Cognitive science research has long established that when reasoning about abstract concepts–such as “nationhood” or “governance”–people commonly resort to knowledge that they have derived from their direct interaction with the world, i.e., things that are perceptually accessible to them through sight, sound, smell, touch, and so on (e.g., [28–34]). Extensive research has found that a mechanism that allows people to reason about abstract ideas in terms of more concrete, experience-based knowledge is conceptual metaphor [33, 35–44]. Many conceptual metaphors are automatically acquired based on everyday experiences, primarily at the early stages of life when basic neural patterns are being formed and strengthened in the mind (e.g., [33, 40, 45, 46]).

For instance, experiencing control commonly co-occurs with experiencing verticality–in a physical struggle, being on top correlates with control, while being at the bottom correlates with lack of control. This experience gives rise to a metaphoric mapping that construes power in terms of verticality, the power-is-up metaphor. Since social power is a rather abstract domain of reasoning that one cannot directly interact with–one cannot touch, smell, or see social power in and of itself–people automatically and largely unconsciously turn to verticality when reasoning about social power [33, 47, 48]. Further, other research finds that conceptual metaphors play a crucial role in the conceptualization of other abstract domains such as divinity, e.g., via the divinity-is-up metaphor [49], morality, e.g., via the morality-is-purity metaphor [50], affection, e.g., via the affection-is-closeness and affection-is-warmth metaphors [51, 52], and even political orientation, e.g., via the conservative-is-right metaphor [53].

Since politics and governance are highly abstract domains of cognition, it comes as no surprise that individuals commonly employ conceptual metaphors when reasoning about politics and forming political attitudes [1, 53–57] (for a review see [35]). Further, there is reason to believe that politicians and advocates implicitly recognize the role of metaphor in political thought, as they commonly employ conceptual metaphors in their discourse [1, 34, 56, 58–61]. And, recent research confirms that the use of political metaphors can be a powerful tool for persuasion [54, 57, 62–65].

In line with this literature, MPT [1] holds that political judgment is largely dependent on conceptual metaphor, and specifically the nation-as-family metaphor. According to MPT, when reasoning about national politics individuals automatically draw on knowledge from their primary and most intimate experiences with group membership and authority: the family. The nation-as-family metaphor serves as a cognitive bridge connecting individuals’ family-level concepts to abstract national-level beliefs. Through the usage of this metaphor, individuals’ beliefs about ideal traits in children and ideal parenting translate into beliefs about ideal traits in citizens and ideal governance. For instance, individuals who ascribe to the strict model’s assertion that parents must severely punish bad behavior in children in order to instill discipline in them will likewise believe in severe punishment for criminals. In support of MPT’s argument, there is evidence that the nation-as-family metaphor is utilized in languages around the world, commonly used by politicians, and play an important role in individuals’ political cognition [1, 56, 59, 60]. For instance, in English, citizenship is commonly construed in terms of family membership (a member of the “American family”), political leaders in terms of heads of the national family (the “founding fathers”), citizens in terms of the family’s children (sending America’s “sons” to war), and nations as homes (the “homeland”). Other languages, besides English, likewise utilize the metaphor, examples include the German “Vaterland” (lit.: father land), which denotes the nation, “Landesväter” (li.: country fathers), which denotes the heads of states, “Vater Staat” (lit.: father state), which denotes the state itself, and “Staatshaushalt” (lit.: state household), which denotes the national budget. Similarly, the Russian “mat' Rossiya” (lit.: mother Russia) means the Russian nation and the Hindi bhaarat maata” (lit.: mother India) denotes the Indian nation. Likewise, political leaders are commonly labeled as the mothers or fathers of a nation. For instance, German chancellor Angela Merkel is called “Mutti Merkel” (lit.: mom Merkel), the first President of the Federal Republic of Germany Theodor Heuss was called “Papa Heuss” (lit.: daddy Heuss), and not least the President of the United States Donald Trump has been labeled “big daddy Trump” [66–69].

## Present research

MPT provides a comprehensive explanation of two fundamental scientific puzzles: Where do political attitudes come from, and why do attitudes on so many seemingly unrelated political issues tend to group together into a left-right divide? For this reason, the theory is highly popular in the cognitive and social sciences and political circles. However, some of the theory’s fundamental claims still lack empirical testing. Importantly, there is no research to date that tests MPT’s assertion that individuals directly map their family ideals onto societal and political attitudes. As described above, some research has explored the correlational relationship between individuals’ family ideals and their political attitudes, but such studies do not test the hypothesized *causal* argument that family ideals directly impact political attitudes. Indeed, past studies leave open the possibility that political attitudes might be the source of family ideals, or that other “third variables” are at the heart of why family ideals and political attitudes correlate with one another. Furthermore, no research has examined the proposed conceptual mechanism by which family ideals map onto societal and political attitudes–i.e., the use of the nation-as-family metaphor, whereby individuals construe nationhood in terms of a family. The present research directly tests these key MPT assertions.

In Studies 1 through 3, we use experimental procedures to explore whether family ideals map onto political attitudes, and to help rule out third variable concerns. Specifically, we manipulate the extent to which participants’ family ideals are cognitively activated prior to them indicating their political attitudes. Then, in Studies 4–5, we test correlationally and experimentally whether the nation-as-family metaphor facilitates the mapping of family beliefs onto political attitudes.

## Study 1

MPT postulates that family ideals map directly onto political attitudes, such that beliefs about how children should behave and how parents should oversee their children directly influence people’s political reasoning and attitudes. To test this hypothesis, in Studies 1 and 2, we manipulated the extent to which the family model that participants most strongly endorse, either strict or nurturant, is cognitively activated in reference to a parenting situation, and then we gauged participants’ attitudes on a political issue in a morally relevant domain. If family models drive participants’ political attitudes, then participants whose parenting beliefs are made particularly salient (manipulated condition) should express more extreme political attitudes in line with that family model as compared to a control condition. To that end, we expected to find more political polarization between strict-endorsers and nurturant-endorsers in the manipulated condition compared to when the two groups were in the control condition.

In Study 1, we presented participants with a common parenting situation: what to do when your baby cries throughout the night even though there is nothing wrong. How one responds in such a situation, either soothing the child or letting him cry himself to sleep, directly reflects which family model an individual ascribes to. Those who endorse the nurturant model will soothe the baby, believing that empathy and nurturance are the right parenting practice. Those who endorse the strict model will let the baby cry himself to sleep, believing in the need to learn self-reliance and that parental indulgence compromises the child’s growth. Along these lines, we presented participants with strict and nurturant responses to the baby-crying scenario, asked them which option they agreed with most, and to elaborate as to why. We hypothesized that strict-endorsers and nurturant-endorsers would demonstrate greater political polarization on political issues in a morally relevant domain (e.g., welfare) in this manipulated condition than those in a control condition not asked about parenting preferences. Specifically, we hypothesized that strict-endorsers in the manipulated condition would support more conservative, and nurturant-endorsers more progressive, positions with regard to welfare and redistribution.

### Materials and methods–Study 1

All research reported below (Studies 1–5) was approved by the University of California, Berkeley’s institutional review board’s Committee for the Protection of Human Subjects (#2011-05-3215).

#### Participants

Four hundred and five participants (201 male, 204 female), recruited from Amazon Mechanical Turk, took part in Study 1 in exchange for a modest payment. We selected this large sample size because we expected, in line with past research [1, 9], that a sizeable portion of our sample would be “biconceptual” (i.e., individuals who endorse both family models, more or less equally). Indeed, past research has shown that approximately 30% of the population is biconceptual [9] (also see [1, 7, 70, 71]). In line with this assumption, we removed any participants from our analyses who indicated that they more strongly endorsed one of the family models (e.g., strict), but subsequently indicated endorsement for the contrasting parenting option (e.g., soothing the crying baby), leaving us with a final total of 304 participants (see S2 File, for further discussion regarding the removal of these participants and for all analyses when including these participants).

#### Procedure

Participants completed the study online through a web interface. The recruitment advertisement indicated that participation would involve partaking in two unrelated studies, one on parenting and one on everyday attitudes.

After completing a short demographic questionnaire, participants indicated whether they would categorize themselves as a strict-endorser or as a nurturant-endorser using a forced-choice item that pilot testing indicated was a robust single-item predictor of participants’ scores on a larger Moral Politics Scale [9]. In particular, participants had to select either “Strictness–parents should be tough toward their children to help ensure their children grow up to be good people,” or “Nurturant–parents should nurture their children’s needs and desires so they can grow up to be whoever they want to be”. Then participants were randomly assigned to either the control or manipulated condition.

In the control condition, participants were asked to think about and describe an average day in their lives. In the manipulated condition participants were presented with a childrearing situation where their baby cries inconsolably even though he is not sick, hungry, or uncomfortable. Then participants were presented with two possible actions that could be taken–one written to directly track a typical nurturant response grounded in empathy and nurturance where the parent picks up and soothes the child, and one written to directly track a typical strict response grounded in immoral indulgence and self-reliance, where the parent does not pick up the child, but instead lets him cry until he calms himself down (see S3 File for full text). After reading these parenting options, participants indicated which one they most strongly agreed with and explained their choice in approximately 5 sentences.

All participants, regardless of condition, learned that they would begin a second (seemingly unrelated) study. Participants then completed 2 questionnaires designed to measure political attitudes on issues that epitomize the contrast between the nurturant ideals of empathy and nurturance and the strict ideals of immoral indulgence and self-reliance at the societal level: the government’s responsibility to ensure citizen’s well-being, and particularly, welfare and redistribution attitudes.

First, participants filled out a 9-item measure of welfare and redistribution attitudes consisting of items taken from an existing measure [72] and items specifically created for this study (see S1 Appendix; e.g., “It’s important we have a solid, well-functioning welfare system.”). The reliability for the 9-items was high (α = .92) so we composited them together to form a Welfare and Redistribution Attitudes Scale. Participants completed these items using a 5-point Likert scale ranging from 1 (strongly disagree) to 5 (strongly agree).

Then, we administered an additional scale gauging beliefs about the relationship between government and citizens [73] asking whether or not a government should, for example, “provide a job for everyone who wants one”, or “provide health care for the sick”. Participants responded to each item on a 5-point scale ranging from 1 (It is definitely NOT the government’s responsibility to…) to 5 (It definitely is the government’s responsibility to…). The reliability across these items was high (α = .89) so we averaged them together to form a Role of Government Scale.

After completing these questionnaires, participants learned that the study was over, and the interface asked them for any comments or thoughts about the “studies” they just took part in. Responses provided no indication that participants thought the two studies were not separate from each other, and no comments suggested that people thought the parenting scenario component had anything to do with the subsequent political attitude questionnaires.

### Results–Study 1

To test our hypothesis that participants in the manipulated condition would become more polarized in their political attitudes than those in the control condition, we conducted 2(family model: strict vs. nurturant) x 2(experimental condition: control vs. manipulated) ANOVAs for each of our dependent variables. Table 1 presents the means and standard deviations for these analysis (see S1 Table for an examination of the effectiveness of random assignment and results when controlling for demographics).

**Table 1:** Means and standard deviations by conditions for participant scores on the *Role of Government* and *Welfare and Redistribution* scales (Study 1).

		Strict	Nurturant	Total
Control	*Role of Government*	*M* = 3.13, *SD* = 1.11	*M* = 3.61, *SD* = 1.00	*M* = 3.45, *SD* = 1.06
	*Welfare and Redistribution*	*M* = 3.14, *SD* = .86	*M* = 3.60, *SD* = .78	*M* = 3.45, *SD* = .83
Manipulated	*Role of Government*	*M* = 2.60, *SD* = 1.10	*M* = 3.81, *SD* = .98	*M* = 3.50, *SD* = 1.14
	*Welfare and Redistribution*	*M* = 2.68, *SD* = .80	*M* = 3.76, *SD* = .74	*M* = 3.49, *SD* = .89
Total	*Role of Government*	*M* = 2.90, *SD* = 1.13	*M* = 3.72, *SD* = .99	
	*Welfare and Redistribution*	*M* = 2.94, *SD* = .86	*M* = 3.69, *SD* = .76	

When entering scores on the Role of Government Scale as the dependent variable, results yielded a significant main effect of family model, *F*(1, 300) = 42.96, *p* < .001, *η*_*p*_^*2*^ = .13, such that nurturant-endorsers scored higher than strict-endorsers. The analysis also found no significant effect of experimental condition *F*(1, 300) = 1.60, *p* = .208, *η*_*p*_^*2*^ = .01. Most relevant for our purposes, the ANOVA yielded a significant interaction, *F*(1, 300) = 7.820, *p* = .006, *η*_*p*_^*2*^ = .03. In line with our hypothesis, there was significantly more polarization in the manipulated condition between strict-endorsers and nurturant-endorsers, *F*(1, 300) = 40.67, *p* < .001, *η*_*p*_^*2*^ = .12 than there was in the control condition between strict-endorsers and nurturant-endorsers, *F*(1, 300) = 7.64, *p* = .006, *η*_*p*_^*2*^ = .02. Furthermore, a simple comparison of the effect of condition for strict-endorsers found that strict-endorsers in the manipulated condition scored significantly higher on the Role of Government Scale than strict-endorsers in the control condition, *F*(1, 300) = 5.83, *p* = .016, *η*_*p*_^*2*^ = .02. A parallel comparison for nurturant-endorsers found a non-significant, though trending, effect of experimental condition, *F*(1, 300) = 2.01, *p* = .158, *η*_*p*_^*2*^ = .01. These results suggest that although the experimental condition caused polarization among strict-endorsers and nurturant-endorsers, the primary explanation for this polarization was that our experimental manipulation had the strongest impact on strict-endorsers.

**Fig 2:**
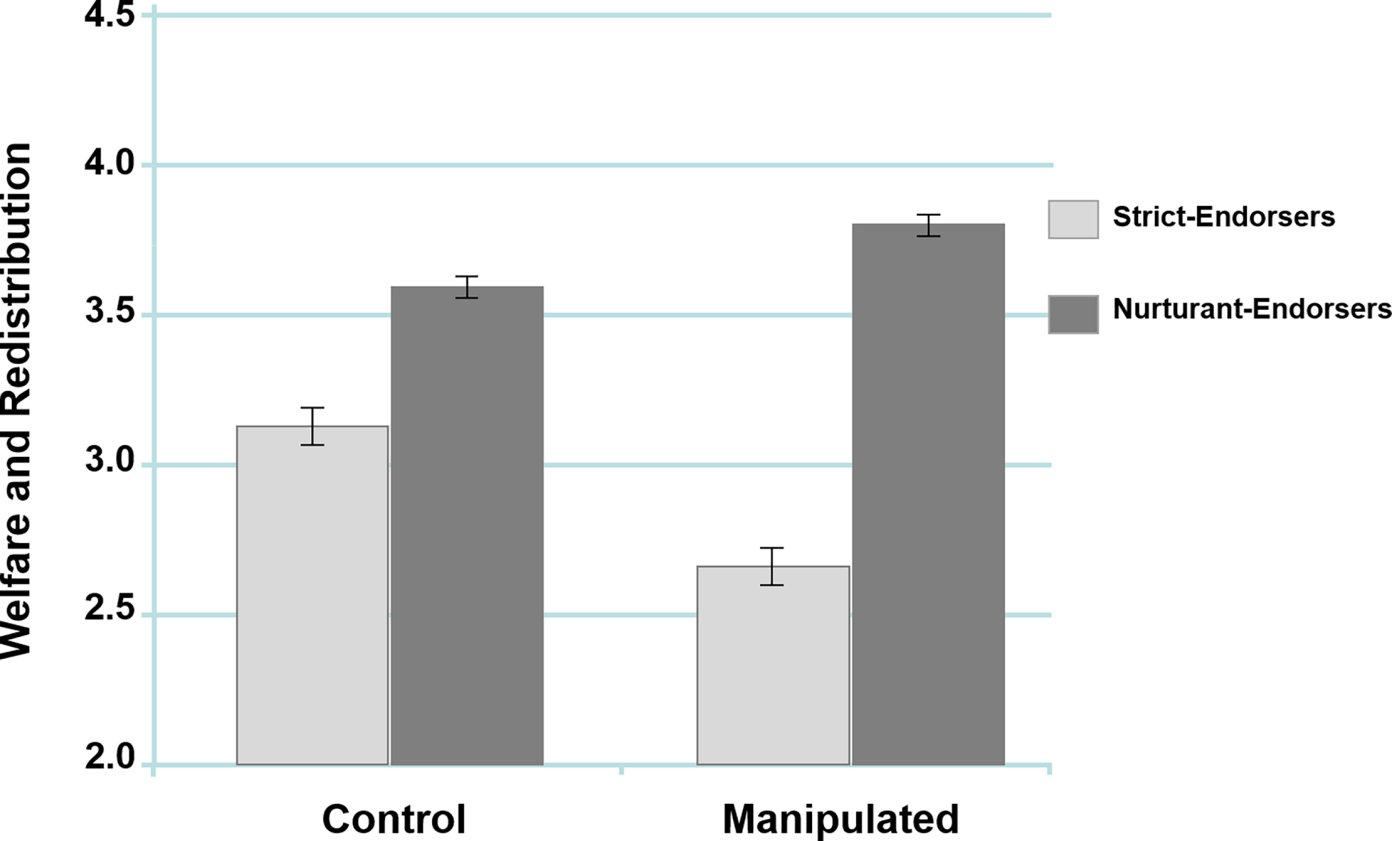
The interaction between ideal family models and experimental condition in predicting welfare and redistribution attitudes. Error bars represent ±1 standard error of the mean (Study 1).

A second 2 x 2 ANOVA entering participants’ scores on the Welfare and Redistribution Attitudes Scale as the dependent variable yielded a significant effect of family model, *F*(1, 300) = 60.88, *p* < .001, *η*_*p*_^*2*^ = .17, with nurturant-endorsers scoring significantly higher than strict-endorsers, *M* = 2.94, *SD* = .86. There was no significant effect of experimental condition, *F*(1, 300) = 2.38, *p* = .124, *η*_*p*_^*2*^ = .01. However, the analysis yielded the predicted family model x experimental condition interaction, *F*(1, 300) = 10.15, *p* = .002, *η*_*p*_^*2*^ = .03 (Fig 2).

As before, there was significantly more political attitude polarization between the strict-endorsers and nurturant-endorsers when in the manipulated condition, *F*(1, 300) = 56.16, *p* < .001, *η*_*p*_^*2*^ = .16, than there was between the strict-endorsers and nurturant-endorsers in the control condition, *F*(1, 300) = 11.52, *p* = .001, *η*_*p*_^*2*^ = .04. Further comparisons within each family model found that there was a significant effect of condition for the strict-endorsers, *F*(1, 300) = .7.91, *p* = .005, *η*_*p*_^*2*^ = .03, but only a trend for the effect of experimental condition on nurturant-endorsers, *F*(1, 300) = 2.31, *p* = .130, *η*_*p*_^*2*^ = .01. Thus, as with the Role of Government Scale, these results suggest that our experimental manipulation had a stronger impact on strict-endorsers than nurturant-endorsers.

### Discussion–Study 1

In Study 1, we asked participants in the manipulated condition to think about and explain their beliefs about what parents should do when their baby continually cries at night even though there is nothing wrong with him. Then, in a seemingly unrelated study, we gauged participants’ attitudes on political issues in a morally relevant political domain (i.e., welfare and redistribution). As hypothesized, we found greater political polarization between strict-endorsers and nurturant-endorsers in the manipulated condition compared to the control condition. These results suggest that activating participants’ beliefs about appropriate parenting subsequently affected their political attitudes, with strict-endorsers becoming more conservative in their positions, and nurturant-endorsers tending to become more progressive in their positions. As such, this first study provides initial support for MPT’s argument that family ideals map directly onto political attitudes.

## Study 2

In Study 2, we aimed to conceptually replicate and extend the findings of Study 1 in various ways. First, we presented a different parenting scenario to the participants in the manipulated condition, this time tapping into beliefs regarding fair treatment and empowerment of children. Specifically, participants read a scenario where 4-year old twin sisters complete chores for a neighbor, but one sister, because she has an attention deficit, completes fewer chores. As a result, even though the twins worked equally hard, the twin who completed fewer chores gets paid less by the neighbor. Again, how one would respond in such a situation, either leveling out the children’s “income” or not, directly reflects which family model an individual ascribes to. Those who endorse the nurturant model will likely level out the children’s rewards, believing that children are best raised on the principles of need-based fairness and empowerment. Those who endorse the strict model, however, will not level out the children’s rewards, believing that children are best raised on the principles of accomplishment-based fairness and competition. As in Study 1, we hypothesized that strict and nurturant participants would demonstrate greater polarization on political issues within a morally relevant domain after the activation of their parenting beliefs.

Additionally, in Study 2, we gauged the effect of our experimental condition on participants’ political attitudes more generally. Although we hypothesized that activating specific aspects of one’s family model (e.g., need-based fairness and empowerment) would strengthen participants’ political attitudes in a morally relevant domain (e.g., social justice policies), we figured that, since the two family models’ different dimensions are internally unified [1, 9], the cognitive activation of one dimension could spread to activate other dimensions. To explore this possibility, we included general measures of political ideology as one of our dependent variables.

### Materials and methods–Study 2

#### Participants

Five hundred and seventy-two participants (263 male, 309 female) recruited from Amazon Mechanical Turk, took part in Study 2 in exchange for a modest payment. We collected a larger sample for this study than in Study 1 for two reasons: First, we wanted to increase statistical power to increase the likelihood that we could find a significant effect of experimental condition between nurturant-endorsers in the manipulated and control conditions–an effect that was only trending, but not significant in Study 1. Second, as in Study 1, we recognized that a portion of the sample would classify as biconceptual. To that end, in Study 2 we removed from analyses participants who more strongly endorsed one of the family models, but subsequently indicated endorsement for the contrasting parenting option, leaving 458 participants (220 male, 238 female) (see S2 File for further discussion regarding the removal of these participants and for all analyses when including these participants). Please also note that discrepancies in degrees of freedom reported in our results were due to some participants not completing all of the dependent measures.

#### Procedure

The procedure for Study 2 was the same as in Study 1. However, instead of a “crying baby” parenting scenario, participants in the manipulated condition read a scenario where their 4-year-old twins both did chores for a neighbor. But because of an attention deficit disorder, one of the twins accomplishes less, and as a result, gets paid less, even though she worked just as hard as the other twin. This discrepancy makes that twin extremely sad and disappointed. Then, these participants read two potential parenting solutions (see S4 File for full text). The first was written to directly track a typical nurturant response grounded in nurturant notions of need-based fairness and empowerment, where the parent gives the child with the attention deficit money to make up for the difference. The second solution was written to directly track a typical strict response grounded in strict notions of accomplishment-based fairness-competition, where the parent does not give the child money to make up for the difference in order to motivate her to do better and discipline herself.

As in Study 1, after reading the parenting solutions, participants indicated which one they most strongly agreed with. Then, they were asked to explain why they thought the selected parenting approach was the right one, and, in addition, on the next screen, they were asked to explain why they believed the approach they did not select to be wrong.

Participants in both conditions learned that they would begin a second, seemingly unrelated study involving political attitude questionnaires. Participants completed 3 questionnaires designed to measure attitudes that utilize the same strict and nurturant dimensions as those present in the twin-sister parenting scenario. Specifically, participants completed the same 5-item Role of Government Scale as in Study 1 [73] (α = .89), as well as a shortened, 5-item version of the Welfare and Redistribution Scale used in Study 1 (α = .91). Additionally, participants completed a 5-item measure of social justice political attitudes (e.g., “We should put more tax money towards empowering people via a good, public education system”; see S1 Appendix). Participants responded to the items on a 5-point Likert scale ranging from 1(strongly disagree) to 5(strongly agree). The reliability for these items was high, α = .80, so we composited them together to form a Social Justice Political Attitudes Scale.

Finally, we included 6 items that measure participants’ political ideology (e.g., “When it comes to politics, to what extent would you consider yourself liberal?”; see S1 Appendix). We z-scored responses to each of these items and then composited them together to form a General Political Attitudes questionnaire (α = .91), with higher scores indicating greater support for progressivism.

After completing these questionnaires, participants learned that the study was over, and they were asked for any comments or thoughts on the “studies” they just partook in. As before, no participants provided comments suggesting that they believed the two studies were not separate or that they believed the parenting scenario component was included as a means of influencing political attitudes.

### Results–Study 2

We conducted a series of 2(family model: strict vs. nurturant) x 2(experimental condition: control vs. manipulated) ANOVAs entering scores on each of our political attitude questionnaires as separate dependent variables. Table 2 presents the means and standard deviations for these analysis (see S2 Table for an examination of the effectiveness of random assignment and results when controlling for demographics).

**Table 2:** Means and standard deviations by conditions for participant scores on the *Role of Government*, *Welfare and Redistribution*, *Social Justice*, and *Political Attitudes* scales (Study 2).

		Strict	Nurturant	Total
Control	*Role of Government*	*M* = 3.34, *SD* = 1.03	*M* = 3.61, *SD* = 1.09	*M* = 3.52, *SD* = 1.08
	*Welfare and Redistribution*	*M* = 3.34, *SD* = .92	*M* = 3.66, *SD* = 1.01	*M* = 3.55, *SD* = .99
	*Social Justice*	*M* = 3.08, *SD* = .70	*M* = 3.40, *SD* = .90	*M* = 3.29, *SD* = .85
	*Political Attitudes*	*M* = -.21, *SD* = .78	*M* = .14, *SD* = .84	*M* = .02, *SD* = .83
Manipulated	*Role of Government*	*M* = 2.99, *SD* = 1.14	*M* = 3.91, *SD* = .90	*M* = 3.49, *SD* = 1.11
	*Welfare and Redistribution*	*M* = 3.03, *SD* = 1.01	*M* = 3.88, *SD* = .77	*M* = 3.49, *SD* = .98
	*Social Justice*	*M* = 2.81, *SD* = .82	*M* = 3.49, *SD* = .80	*M* = 3.19, *SD* = .87
	*Political Attitudes*	*M* = -.39, *SD* = .86	*M* = .27, *SD* = .73	*M* = -.03, *SD* = .85
Total	*Role of Government*	*M* = 3.16, *SD* = 1.10	*M* = 3.73, *SD* = 1.03	
	*Welfare and Redistribution*	*M* = 3.18, *SD* = .98	*M* = 3.75, *SD* = .94	
	*Social Justice*	*M* = 2.94, *SD* = .77	*M* = 3.44, *SD* = .86	
	*Political Attitudes*	*M* = -.30, *SD* = .82	*M* = .19, *SD* = .80	

**Fig 3:**
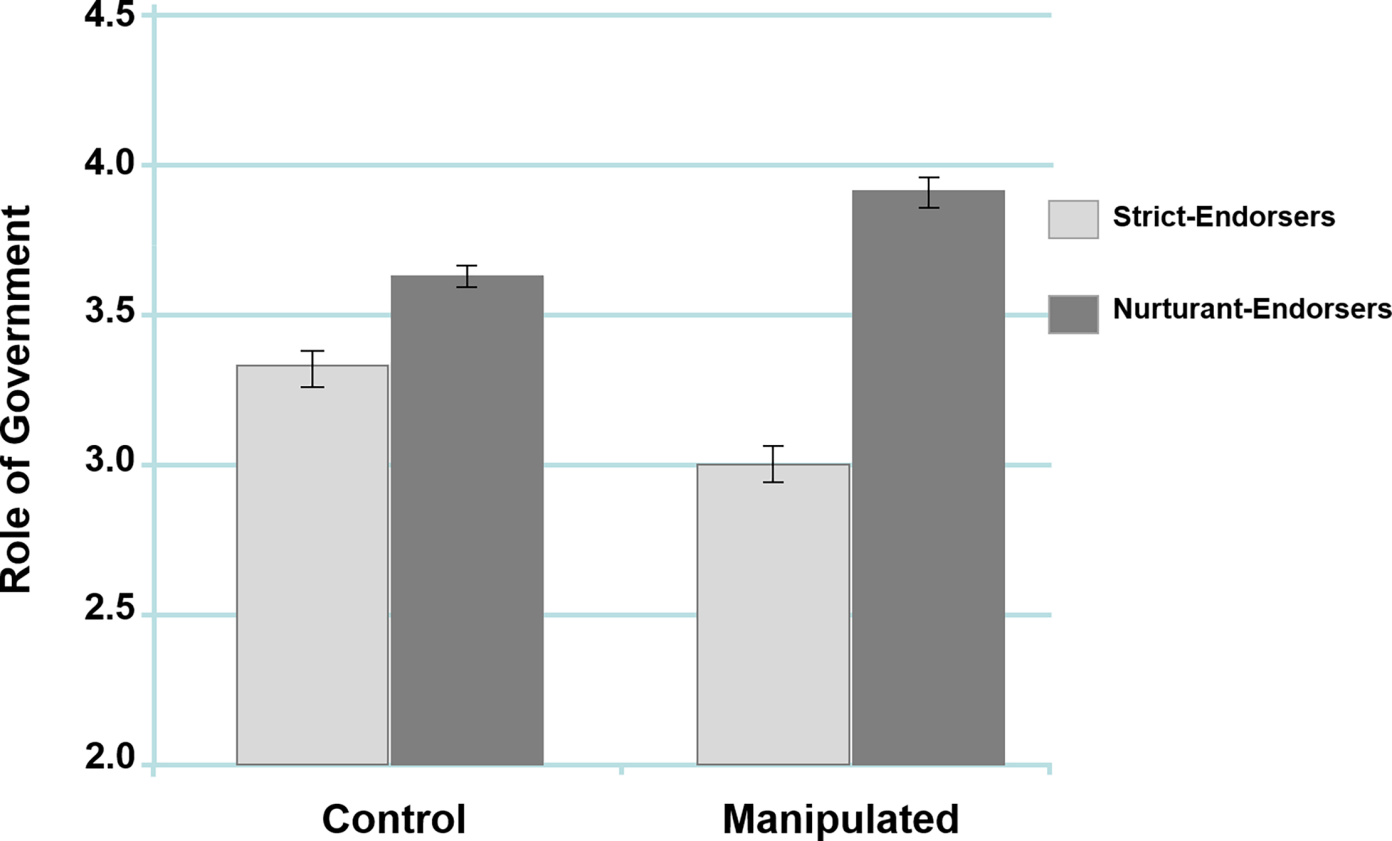
The interaction between ideal family models and experimental condition in predicting attitudes about the role of government. Error bars represent ±1 standard error of the mean (Study 2).

For the Role of Government Scale, we found a main effect of family model, *F*(1, 447) = 34.59, *p* < .001, *η*_*p*_^*2*^ = .07, such that nurturant-endorsers scored higher than strict-endorsers, further verifying that the two family models, regardless of experimental condition, are robust predictors of political attitudes. The analysis also found a non-significant effect of experimental condition, *F*(1, 447) < 1, *p* < .777, *η*_*p*_^*2*^ < .01, as well as the predicted 2 x 2 interaction, *F*(1, 447) = 10.17, *p* = .002, *η*_*p*_^*2*^ = .02 (Fig 3).

In line with our hypothesis, there was significantly more polarization in the manipulated condition between strict-endorsers and nurturant-endorsers, *F*(1, 447) = 39.09, *p* < .001, *η*_*p*_^*2*^ = .08, than there was in the control condition between strict-endorsers and nurturant-endorsers, *F*(1, 447) = 3.83, *p* = .05, *η*_*p*_^*2*^ = .01. Additionally, comparisons within each family model found that strict-endorsers in the manipulated condition scored significantly lower than those in the control condition, *F*(1, 447) = 5.02, *p* = .026, *η*_*p*_^*2*^ = .01, and that nurturant-endorsers in the manipulated condition scored significantly higher than those in the control condition, *F*(1, 447) = 5.29, *p* = .022, *η*_*p*_^*2*^ = .01. These results suggest that the greater political polarization that we found in the manipulated condition (relative to the control) was due to both strict-endorsers and nurturant-endorsers becoming more extreme in their political attitudes.

For welfare and redistribution attitudes, the 2 x 2 ANOVA yielded a significant effect of family model, *F*(1, 449) = 40.80, *p* < .001, *η*_*p*_^*2*^ = .08, with nurturant-endorsers scoring higher than strict-endorsers. There was no significant difference due to experimental condition, *F*(1, 449) = .23, *p* = .630, *η*_*p*_^*2*^ < .01, but there was a significant 2 x 2 interaction, *F*(1, 449) = 7.96, *p* = .005, *η*_*p*_^*2*^ = .02. As before, there was significantly more polarization in the manipulated condition between strict-endorsers and nurturant-endorsers, *F*(1, 449) = 40.33, *p* < .001, *η*_*p*_^*2*^ = .08, than there was in the control condition between strict-endorsers and nurturant-endorsers, *F*(1, 449) = 6.70, *p* = .010, *η*_*p*_^*2*^ = .01. Furthermore, we found that for strict-endorsers there was a significant effect of condition, *F*(1, 449) = 4.53, *p* = .034, *η*_*p*_^*2*^ = .01 and for nurturant-endorsers there was a marginally significant effect of condition, *F*(1, 449) = 3.44, *p* = .064, *η*_*p*_^*2*^ = .01.

The 2 x 2 ANOVA entering Social Justice Political Attitudes as the dependent variable yielded a significant effect of family model, *F*(1, 454) = 39.77, *p* < .011, *η*_*p*_^*2*^ = .08. Nurturant-endorsers scored significantly higher than did strict-endorsers. The analysis yielded a non-significant effect of experimental condition, *F*(1, 454) = 1.32, *p* = .251, *η*_*p*_^*2*^ < .01, and a significant interaction, *F*(1, 454) = 5.03, *p* = .025, *η*_*p*_^*2*^ = .01. In the manipulated condition, there was significantly more polarization between the strict-endorsers and nurturant-endorsers, *F*(1, 454) = 34.79, *p* < .001, *η*_*p*_^*2*^ = .07, than there was in the control condition between strict-endorsers and nurturant-endorsers, *F*(1, 454) = 8.70, *p* = .003, *η*_*p*_^*2*^ = .02. An examination of the effect of experimental condition within each family model group found that strict-endorsers in the manipulated condition scored significantly lower than strict-endorsers in the control condition, *F*(1, 454) = 4.75, *p* = .030, *η*_*p*_^*2*^ = .01. However, there was no significant difference due to condition for nurturant-endorsers, *F*(1, 454) = .76, *p* = .385, *η*_*p*_^*2*^ < .01.

Finally, to explore whether our experimental manipulation might have a more general effect on political attitudes, we conducted an additional 2 x 2 ANOVA entering participants’ scores on the General Political Attitudes scale as the dependent variable. We found a significant difference due to family model, *F*(1, 446) = 40.89, *p* < .001, *η*_*p*_^*2*^ = .08. Nurturant-endorsers scored significantly higher (more progressive) than strict-endorsers. As with the above analyses, there was no significant effect of experimental condition, *F*(1, 446) < 1, *p* = .772, *η*_*p*_^*2*^ < .01, but there was a significant interaction, *F*(1, 446) = 4.11, *p* = .043, *η*_*p*_^*2*^ = .01. An examination of the simple effects revealed that there was greater polarization in the manipulated condition between strict-endorsers and nurturant-endorsers, *F* (1, 446) = 33.60, *p* < .001, *η*_*p*_^*2*^ = .07, than there was in the control condition between strict-endorsers and nurturant-endorsers, *F*(1, 446) = 10.10, *p* = .002, *η*_*p*_^*2*^ = .02. An examination of the effect of condition within each family model group found that the effect of condition on strict-endorsers was in the right direction but not significant, *F*(1, 446) = 2.23, *p* = .136, *η*_*p*_^*2*^ < .01, and likewise was in the right direction for nurturant-endorsers but not significant, *F*(1, 446) = 1.90, *p* = .169, *η*_*p*_^*2*^ < .01.

### Discussion–Study 2

In Study 2, we had participants in the manipulated condition think about and explain their attitudes regarding a parenting situation designed to trigger parenting beliefs about what constitutes fair treatment of children and, relatedly, whether children should be empowered or left to compete. We hypothesized that strict-endorsers and nurturant-endorsers in this condition, relative to a control, would subsequently indicate more extreme political attitudes on a morally relevant political domain (e.g., government assistance), with nurturant-endorsers espousing more progressive and strict-endorsers more conservative attitudes. Overall, we found consistent support for this hypothesis. We also found that our experimental manipulation had a polarizing effect on strict-endorsers’ and nurturant-endorsers’ general political attitudes, even though these attitudes were not directly based on a morally relevant political domain. Altogether, these results provide further evidence for MPT’s assertion that individuals’ parenting beliefs drive their political attitudes.

## Study 3

Asking participants to think about and explain their parenting beliefs in Studies 1 and 2 resulted in strict-endorsers espousing more extreme conservative, and nurturant-endorsers more extreme progressive, attitudes. We interpreted these results as evidence that family model beliefs play a fundamental role in dictating political attitudes. However, one might alternatively interpret our results as having nothing to do with family models specifically, but rather as an example of general concept priming. In our studies, we asked participants who identified as nurturant or strict to think about childrearing situations involving being nurturant or strict, respectively. Thus, it is conceivable that we primed these individuals with general beliefs or identities related to nurturance or strictness, and not necessarily beliefs specific to family life. This more general priming could, in turn, have led to the more extreme political attitudes. If so, this would suggest that the relationship between individuals’ family models and political attitudes is not a causal one, but rather driven by an overarching concept, acting as a “third variable” that links family models to political attitudes.

Further, in Studies 1 and 2, we told participants that the parenting and political attitudes components were separate studies, and participants gave no indication of believing otherwise. Yet, it is possible that Study 1’s and 2’s procedures created a demand effect, such that participants recognized that we were exploring the relationship between parenting beliefs and political attitudes. In response, participants may have expressed more extreme progressive and conservative attitudes, believing that was what the experimenters wanted them to do [74].

To address both of these concerns, in Study 3 we replicated Study 2’s procedure, but “in reverse”. Specifically, we asked participants whether they identified more strongly as progressive or conservative, and then presented them with a scenario describing the plight of individuals who live impoverished lives in America. We then asked participants which of two policy stances relating to government assistance they believed to be more appropriate, and had them explain why. Next, we gauged their attitudes on a morally relevant parenting situation, as well as their larger strict and nurturant parenting beliefs, to see if making arguments in favor of one’s morally strict or nurturant political attitudes influences one’s parenting beliefs.

If the parenting scenarios from Studies 1 and 2 were just serving to prime a more general third variable (e.g., strictness, self-reliance, kindness, compassion), then we would expect that asking participants to explain their political attitudes on a morally relevant issue would prime this same third variable, and, as a result, lead them to express more extreme family beliefs: Asking conservatives to explain their political stance would drive them to be more extreme strict-endorsers in the family domain, and asking progressives to explain their political stance would push them to be more extreme nurturant-endorsers in the family domain. However, if we do not find that asking participants to explain their political stances affects parenting beliefs, then that would provide support for MPT’s claim that parenting beliefs–in particular–are a fundamental driver of political attitudes.

Additionally, if the results in Studies 1 and 2 were due to demand effects because participants in the manipulated condition inferred the studies’ hypotheses, then we should expect the same recognition to occur when the study is run in reverse. Thus, we should find greater polarization in family beliefs for those participants in the manipulated condition. However, if we do not find significant effects when the study is run in reverse, it would suggest that our findings in Studies 1 and 2 were not due to a demand effect.

### Materials and methods–Study 3

#### Participants

Five hundred and four participants (225 male, 278 female, 1 did not indicate), recruited from Amazon Mechanical Turk, took part in Study 1 in exchange for a modest payment. We found that 48 participants (10%) indicated they were progressive or conservative, but subsequently indicated support for the contrasting political position–i.e., they were biconceptuals. These participants were removed from analyses, leaving 456 participants (210 male, 246 female). With this sample size, we estimated that we should be able to find a significant 2(political ideology: progressive vs. conservative) x 2(experimental condition: control vs. manipulated) interaction if a small effect existed (i.e., *f* ~ .13) at .80 power. Please also note that discrepancies in degrees of freedom reported in our results were due to some participants not completing all of the dependent measures.

#### Procedure

Like in Studies 1 and 2, the recruitment advertisement indicated that the participants would partake in two unrelated studies. As part of a demographic questionnaire, participants indicated whether they were “liberal/leaning liberal” or “conservative/leaning conservative”. Then, participants were randomly assigned to either a control or manipulated condition. As in Studies 1 and 2, participants in the control condition wrote about a typical day in their lives, while those in the manipulated condition read a paragraph describing how hard it is, even for those who work extremely hard, to achieve financial and social success in America (see S5 File for full text of all stimuli). Then participants read two policy arguments to help solve this problem, one typically progressive and one typically conservative. The typically progressive argument emphasized that the government should provide public assistance programs, whereas the typically conservative argument emphasized treateing everyone equally and therefore keeping the government from getting involved. Participants indicated which of the two arguments they most strongly agreed with and explained why their selected argument was right and the other argument was wrong.

All participants then learned that they would begin the second, seemingly unrelated study focusing on parenting beliefs. Participants answered 3 questionnaires. The first was a 5-item Parenting Siblings measure designed to gauge participants’ beliefs regarding (nurturant) need-based and (strict) accomplishment-based fairness in raising siblings. Example items included “Even if one sibling is doing worse than the other, parents should not provide extra help to the child who is failing. Rather, that child needs to become self-sufficient and learn how to succeed on his or her own” and “It’s good for children to learn early that failure is a signal that one must work harder” (α = .70). Participants responded on a 5-point scale, ranging from 1 (strongly disagree) to 5 (strongly agree), with higher scores indicating greater support for strict beliefs (see S1 Appendix for all items).

Next, participants completed the two subscales of the Moral Politics Scale [9]. The first measured participants’ strict parenting beliefs (e.g., “‘Tough love’ is required to raise a child right”; α = .87). The second subscale measured participants’ nurturant parenting beliefs (e.g., “In order to truly nurture children one needs to be empathic”; α = .86). Participants responded to each item using a 7-point scale ranging from 1(strongly disagree) to 7 (strongly agree) (see S1 Appendix for all items).

After completing these questionnaires, participants learned that the study was over. We asked them for any comments or thoughts they had about the “studies”. As in Studies 1 and 2, no participants’ comments suggested that they believed the two studies were actually not separate, and no participants indicated they believed the political component of the study was used as a means of influencing parenting beliefs.

### Results–Study 3

We conducted a series of 2(political ideology: progressive vs. conservative) x 2(experimental condition: control vs. manipulated) ANOVAs entering scores on each of our parenting beliefs questionnaires as separate dependent variables (see S3 Table for an examination of the effectiveness of random assignment and results when controlling for demographics). For the Parenting Siblings scale, the analysis yielded a significant main effect of political ideology, *F*(1, 451) = 10.19, *p* = .002, *η*_*p*_^*2*^ = .02, with conservative participants scoring higher, *M* = 2.81, *SD* = .63, than progressive participants, *M* = 2.61, *SD* = .64. There was no main effect of experimental condition, *F*(1, 451) = 1.94, *p* = .164, *η*_*p*_^*2*^ < .01. Likewise, there was no interaction, *F*(1, 451) = 1.45, *p* = .229, *η*_*p*_^*2*^ < .01.

When entering strict parenting beliefs as the dependent variable, we found a significant effect of political ideology, *F*(1, 450) = 34.26, *p* < .001, *η*_*p*_^*2*^ = .07, such that conservatives scored significantly higher, *M* = 4.90, *SD* = .73, than progressives, *M* = 4.42, *SD* = .88. There was no main effect of experimental condition, *F*(1, 450) < 1, *p* = .90, *η*_*p*_^*2*^ < .01, and we found no interaction, *F*(1, 450) < 1, *p* = .84, *η*_*p*_^*2*^ < .01. Similarly, when we entered nurturant parenting beliefs as the dependent variable, the analysis yielded a significant main effect of ideology, *F*(1, 450) = 12.89, *p* < .001, *η*_*p*_^*2*^ = .03, with progressives scoring significantly higher, *M* = 5.49, *SD* = .83, than conservatives, *M* = 5.21, *SD* = .73. But we found no main effect of condition, *F*(1, 450) < 1, *p* = .941, *η*_*p*_^*2*^ < .01, and no interaction, *F*(1, 450) < 1, *p* = .578, *η*_*p*_^*2*^ < .01.

### Discussion–Study 3

In Study 3, to rule out that our findings from Studies 1 and 2 were due to priming an overarching concept rather than triggering parenting beliefs in particular, we conducted a reverse version of Study 2. We asked progressive and conservative participants to think about and explain their stance on government assistance to less prosperous individuals and then, in a seemingly unrelated study, we asked participants about their parenting beliefs on a morally relevant topic (i.e., what to do when children do not achieve equal levels of some “objective” measure of success) as well as their more general strict and nurturant beliefs. Unlike in Studies 1 and 2, we found no significant interactions–progressives and conservatives did not become more extreme in their parenting beliefs when their political beliefs were activated.

The results in Study 3 suggest that the effects found in Studies 1 and 2 were not due to the priming of a more general (not family-related) concept, since this same concept would have also been primed in Study 3. Further, Study 3’s design helps rule out the possibility that the effects we found in Studies 1 and 2 were due to demand effects, such that participants recognized our research hypothesis and then acted to support it. Specifically, there is little reason to believe that participants in our first two studies realized that we were examining carryover effects of parenting beliefs to political attitudes, while participants in Study 3 did not realize that we were examining carryover effects of political attitudes to parenting beliefs. Altogether then, the results of Study 3 help address alternate explanations for the results found in Studies 1 and 2, and therefore provide further support for MPT’s argument that parenting beliefs directly map onto political attitudes.

## Study 4

MPT asserts that people map their family beliefs onto the political domain by engaging the nation-as-family metaphor. In Studies 4 and 5 we tested this assertion. In Study 4 we tested the metaphor’s role by exploring whether the extent to which individuals overtly engage the metaphor moderated the relationship between parenting beliefs and political stances. Although MPT asserts that the parenting-politics link occurs through the nation-as-family metaphor for all individuals, there are likely differences in how much individuals overtly recognize and utilize the metaphor. For instance, the metaphor might play a more prominent role for individuals whose everyday lives are closely entrenched with their nuclear families, who regularly think about and discuss politics, and who commonly use the nation-as-family mapping when doing so. To that end, in line with MPT, those who engage the metaphor most overtly and explicitly should demonstrate the strongest link between parenting beliefs and political ideology.

Additionally, in Study 4, we more closely explored the conceptual path by which parenting beliefs directly map onto political attitudes. MPT asserts that in order for individuals to reason about larger social groups with authorities, they resort to their idealized parenting models as templates. It is through this process that family beliefs, in turn, map directly onto political attitudes. To examine this mediating role of moral societal attitudes, in Study 4, we measured participants’ nurturant and strict societal beliefs along with measuring their parenting beliefs and political attitudes.

### Materials and methods–Study 4

#### Participants

Two hundred and one participants (100 male, 101 female), recruited from Amazon Mechanical Turk, took part in Study 4 in exchange for a modest payment.

#### Procedure

The study involved filling out a series of questionnaires, including some that served as distractors/fillers to avoid creating demand effects and help ensure that relationships between similar questionnaires were not due to consistency effects.

Participants first completed the two subscales of the Moral Politics Scale [9] that we used in Study 3 (α_strict_ = .85; α_nurturant_ = .85). Then, participants completed a 5-item measure that gauged the extent to which they overtly engage the nation-as-family metaphor (e.g., “Although the citizens of one nation are not all related to each other, they are in a way ‘part of one big family’”; see S1 Appendix for all items). Participants responded to these using a 7-point scale ranging from 1(strongly disagree) to 7(strongly agree). The reliability among the 5 items was high (α = .87), so we averaged them together to form a Nation-as-Family Metaphor Engagement Questionnaire.

Following this, participants completed two questionnaires unrelated to parenting beliefs or political attitudes that we included as distractors/fillers. Together, these questionnaires involved responding to approximately 40 items and took about five minutes to complete. Then, participants completed a measure to gauge their nurturant and strict societal beliefs, the Moral Society Scale, which we modeled after the Moral Politics Scale [9]. We developed this scale in order to capture participants’ beliefs about how society should function as a whole using a strict and nurturant framework. The scale included “citizen” and “governance” versions of each item of the Moral Politics Scale. Specifically, wherever an item in the Moral Politics Scale referred to “children”, we replaced this with “citizens”, “the public”, or conceptually similar words. Likewise, wherever an item referred to “parents”, we replaced this with “the government”, “national authorities”, or conceptually similar words. For example, the strict item “Obedience must be instilled in children” became “The government must instill obedience in its citizens” and the nurturant item “Siblings should receive parental support in accordance to their individual needs” became “Americans should receive governmental assistance in accordance to their individual needs” (α_strict society_ = .82; α_nurturant society_ = .81; see S1 Appendix for the Moral Society Scale).

Finally, participants completed a 4-item version of the political ideology measure used in Study 2, which involved participants indicating the extent to which they identified as progressive and conservative and the extent to which they felt warm or cold toward progressives and conservatives (see S1 Appendix for all items). We composited these items together to form a political ideology measure (α = .96) with higher scores indicating greater progressivism. After completing these measures, participants filled out a demographic questionnaire and then learned that the study was over.

### Results and discussion–Study 4

Scores on the strict subscale were subtracted from those on the nurturant subscale to create a single measure of parenting beliefs, such that higher scores indicated greater endorsement of nurturant attitudes relative to strict attitudes. Likewise, we subtracted strict from nurturant moral society scores to create a single measure of moral society beliefs, with higher scores indicating stronger support for nurturant relative to strict beliefs. Table 3 presents the zero-order correlations among all of our variables.

**Table 3:** Zero-order correlations among subscales from the Moral Politics Scale, the Moral Society Scale, metaphor endorsement, and political attitudes. Numbers in brackets represent the 95% confidence interval (Study 4).

	1.	2.	3.	4.	5.	6.
1. Strict Family		.00 [.00 ,.08]	.58*** [.47,.68 ]	-.06 [-.24,.12]	.00 [.00,.08 ]	-.20** [-.33,-.07]
2. Nurturant Family			-.13 [-.28,.02 ]	.69*** [.59,.77]	.21** [.05,.34]	.26*** [.15,.38]
3. Strict Moral Society				-.02 [-.19,.14]	.34*** [.20,.48]	-.06 [-.20,.07]
4. Nurturant Moral Society					.35*** [.21,.47]	.38*** [.25,.49]
5. Metaphor Endorsement						.18* [.02,.32]
6. Political Attitudes						

Note

* indicates p < .05

** indicates p < .01

*** indicates p < .001.

To examine the role of the nation-as-family metaphor in linking participants’ family models to their political attitudes, we first ran a multiple regression analysis entering participants’ scores on the Moral Politics Scale, the Nation-as-Family Metaphor Engagement Questionnaire, and the interaction of the two as predictors of political attitudes. This analysis yielded a significant main effect of Moral Politics Scale, *b* = .25, *S*.*E*. = .05, *p* < .001, indicating that the more individuals endorse the nurturant relative to the strict parenting model, the more progressive they tend to be (see S6 File for results when controlling for demographics). We also found a non-significant effect of Nation-as-Family Metaphor Engagement Questionnaire, *b* = .11, *S*.*E*. = .06, *p* = .063, and, most importantly, a significant interaction, *b* = .10, *S*.*E*. = .05, *p* = .028. Simple slope analyses indicated that at lower (-1 SD) endorsement of the nation-as-family metaphor, scores on the Moral Politics Scale were weaker predictors of political ideology, *b* = .14, *S*.*E*. = .07, *p =* .045, than they were for high endorsers (+1 SD) of the nation-as-family metaphor, *b* = .35, *S*.*E*. = .07, *p* < .001. Such a result suggests that the nation-as-family metaphor facilitates the link between parenting beliefs and political attitudes–the more individuals overtly endorsed the metaphor, the stronger the link between their parenting models and their political attitudes.

We conducted a parallel regression analysis, but this time entered scores on the Moral Society Scale as the dependent variable. This analysis yielded a significant main effect of Moral Politics Scale, *b* = .76, *S*.*E*. = .05, *p* < .001, such that the more one endorses the nurturant relative to the strict parenting model, the more one also endorses nurturant relative to strict moral societal beliefs. Additionally, we found an unexpected effect of the Nation-as-Family Metaphor Engagement Questionnaire, *b* = -.13, *S*.*E*. = .06, *p* = .025, suggesting that the more individuals overtly and explicitly endorse the nation-as-family metaphor, the less they endorse nurturant relative to strict moral society beliefs. Most relevant to testing our hypothesis, we found a significant interaction, *b* = .09, *S*.*E*. = .04, *p* = .036. For low endorsers (-1 SD) of the nation-as-family metaphor, Moral Politics Scale scores were a weaker predictor of Moral Society Scale scores, *b* = .67, *S*.*E*. = .07, *p* < .001, than they were for those scoring high (+1 SD) on endorsement of the nation-as-family metaphor, *b* = .86, *S*.*E*. = .07, *p* < .001. This result suggests that the nation-as-family metaphor facilitates the connection between parenting beliefs and societal attitudes.

To examine whether the parenting models map onto political attitudes through societal-level beliefs, and whether the nation-as-family metaphor facilitates each step of this path, we next conducted a mediated moderation analysis [75], entering political ideology as the dependent variable, Moral Politics Scale scores as the predictor, Nation-as-Family Metaphor Engagement Questionnaire scores as the moderator, and Moral Society Scale scores as the mediator. A bootstrap analysis with 5000 samples revealed that the 95% confidence interval for the indirect effect went from -.003 to .031, indicating that the mediated moderation model was marginally significant. Even so, this result provides some evidence that parenting beliefs map onto political attitudes because individuals rely on idealized family models when reasoning about how society should work, and, in turn, derive their policy stances from those moral societal models. Additionally, this marginal effect suggests that the nation-as-family metaphor likely facilitates the conceptual path from parenting to society to politics.

## Study 5

In Study 5, we manipulated endorsement of the nation-as-family metaphor and examined the effects this manipulation had on the relationship between individuals’ strict and nurturant parenting beliefs and their political attitudes. We hypothesized that participants led to not actively utilize the metaphor would demonstrate a weaker relationship between parenting beliefs and political attitudes than participants led to actively utilize the metaphor.

Specifically, we presented participants with an argument either explaining why the nation-as-family metaphor is highly applicable or why the metaphor makes little sense. We predicted that weakening or strengthening the likelihood with which participants explicitly utilized the metaphor would impact the extent to which they rely on the metaphoric mapping for political cognition. Decreasing participants’ active utilization of the metaphor, we figured, would weaken the relationship between parenting beliefs and political attitudes, whereas increasing their utilization of the metaphor would strengthen it. Thus, in the metaphor-decrease condition, we expected to find less political polarization between strict-endorsers and nurturant-endorsers than in the metaphor-increase condition.

### Materials and methods–Study 5

#### Participants

Six hundred participants (279 male, 320 female, 1 did not indicate), recruited from Amazon Mechanical Turk, took part in Study 5 in exchange for a modest payment.

#### Procedure

As in Studies 1 and 2, participants completed a questionnaire that asked them to indicate whether they more strongly endorsed a nurturant or strict parenting model, using the strictness-versus-nurturant item (Importantly, family values were not measured via the Moral Politics Scale, because, as described below, the metaphor manipulation involved asking participants to think about how the family domain metaphorically maps (or does not map) onto the socio-political domain. Having participants fill out the Moral Politics Scale prior to this manipulation would likely create a demand effect). Then, they were randomly assigned to either a metaphor-increase or metaphor-decrease condition. Participants in the metaphor-increase condition were told about the nation-as-family metaphor and how accurate and common this metaphor is, and were then instructed to think of and list ways in which the nation is just like a family. Participants in the metaphor-decrease condition were told about the metaphor, but were informed about how silly and inadequate it is. They were then asked to think of and list ways in which the nation is not like a family at all (see S7 File for full text).

After completing this task, participants indicated their agreement with the following statement: “In many ways, governing a nation is like running a family,” using a 7-point scale ranging from 1 (strongly disagree) to 7 (strongly agree). This served as a means for measuring the extent to which the manipulation increased or decreased participants’ endorsement of the nation-as-family metaphor.

Participants then completed the Moral Society Scale from Study 4. As before, reliability on both the strict and nurturant society subscales was high (α_strict society_ = .83; α_nurturant society_ = .85). Finally, participants completed a 10-item measure of “hot-button” political attitudes (e.g., gay marriage, abortion, and tax policy; see S1 Appendix for all items). Participants indicated the extent to which they agreed or disagreed with each political stance on a 7-point scale, ranging from 1(strongly against) to 7(strongly in favor). All items were scored so that higher scores indicated greater support for progressive stances (α = .79). Once participants had completed this questionnaire, the study was over.

### Results–Study 5

#### Manipulation check

To test whether exposure to the metaphor-increase or metaphor-decrease condition influenced usage of the nation-as-family metaphor, we conducted a *t*-test comparing the extent to which participants in each condition agreed with the manipulation check item. This analysis yielded a significant difference, *t*(596) = 18.12, *p* < .001, such that participants in the metaphor-increase condition reported greater agreement, *M* = 4.75, *SD* = 1.53, and participants in the metaphor-decrease condition reported less agreement, *M* = 2.57, *SD* = 1.41. As such, we concluded that the manipulation successfully influenced participants’ active utilization of the nation-as-family metaphor.

Next, we examined the possibility that the experimental manipulation might have influenced participants espousing strict and nurturant beliefs differently. To rule out this possibility, a 2(parenting model: strict versus nurturant) x 2(metaphor condition: increase versus decrease) ANOVA was conducted, entering scores on the manipulation check item as the dependent variable. This analysis yielded no significant interaction between family ideals and metaphor condition, *F*(1, 594) < 1, *p* = .639, *η*_*p*_^*2*^ < .01, suggesting that the metaphor manipulation did not affect strict-endorsers and nurturant-endorsers in different ways.

#### Moral society attitudes

Scores on the strict society scale were subtracted from those on the nurturant society subscale to create a single measure of Moral Society attitudes, so that higher scores indicated greater endorsement of nurturant relative to strict attitudes. We conducted a 2(parenting model: strict versus nurturant) x 2(metaphor condition: increase versus decrease) ANOVA, entering Moral Society Scale scores as the dependent variable (see Table 4 for means and standard deviations; (see S4 Table for an examination of the effectiveness of random assignment and results when controlling for demographics).).

**Table 4:** Means and standard deviations by conditions for participant scores on the *Moral Society*, and *Political Attitudes* scales (Study 5).

		Strict	Nurturant	Total
Metaphor Increase	*Moral Society*	*M* = .14 *SD* = 1.16	*M* = 1.38 *SD* = 1.23	*M* = .96, *SD* = 1.34
	*Political Attitudes*	*M* = 4.11, *SD* = 1.09	*M* = 5.09, *SD* = .96	*M* = 4.76, *SD* = 1.10
Metaphor Decrease	*Moral Society*	*M* = .47 *SD* = 1.08	*M* = 1.22 *SD* = 1.29	*M* = .99, *SD* = 1.28
	*Political Attitudes*	*M* = 4.28, *SD* = 1.10	*M* = 4.81, *SD* = 1.11	*M* = 4.65, *SD* = 1.13
Total	*Moral Society*	*M* = .30, *SD* = 1.13	*M* = 1.30, *SD* = 1.27	
	*Political Attitudes*	*M* = 4.20, *SD* = 1.10	*M* = 4.94, *SD* = 1.05	

The analysis yielded a significant main effect of family model endorsement, *F*(1, 596) = 86.85, *p* < .001, *η*_*p*_^*2*^ = .13, such that those endorsing strict beliefs scored significantly lower than those endorsing nurturant beliefs. The analysis also yielded a non-significant effect of the experimental condition, *F*(1, 596) < 1, *p* = .407, *η*_*p*_^*2*^ < .01. Further, we found the predicted significant interaction, *F*(1, 596) = 5.13, *p* = .024, *η*_*p*_^*2*^ = .01. Strict-endorsers and nurturant-endorsers in the metaphor-increase condition were significantly more polarized, *F*(1, 596) = 67.61, *p* < .001, than in the metaphor-decrease condition, *F*(1, 596) = 24.70, *p* < .001. However, when examining differences within each family model, we found that our manipulation led to only a marginal difference for strict-endorsers, *F*(1, 596) = 3.50, *p* = .062, *η*_*p*_^*2*^ = .01, and a non-significant difference for the nurturant-endorsers, *F*(1, 596) = 1.62, *p* = .203, *η*_*p*_^*2*^ < .01.

#### Political attitudes

**Fig 4:**
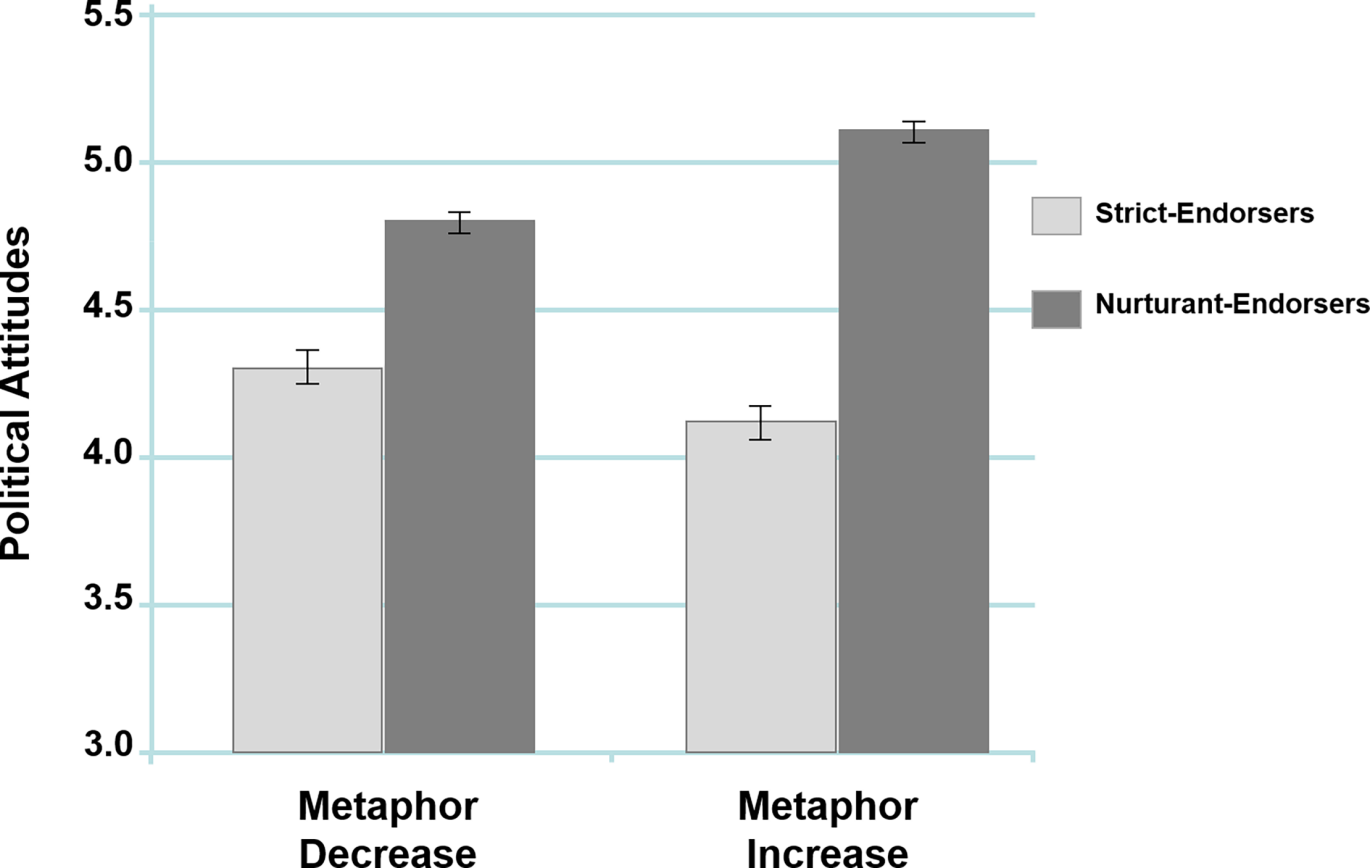
Effects of the metaphor-increase and metaphor-decrease conditions on strict and nurturant policy attitudes. Error bars represent ±1 standard error of the mean (Study 5).

We conducted the same 2(parenting model: strict versus nurturant) x 2(metaphor condition: increase versus decrease) ANOVA, this time entering scores on the political attitudes questionnaire as the dependent variable (see Table 4 for means and standard deviations). This analysis yielded a significant main effect of family model endorsement, *F*(1, 596) = 65.01, *p* < .001, *η*_*p*_^*2*^ = .10, such that those espousing nurturant ideals demonstrated greater progressive policy support than those espousing strict ideals. Also, there was a non-significant effect of condition, *F*(1, 596) < 1 , *p* = .565, and we found a significant interaction, *F*(1, 596) = 5.64, *p* = .018. As shown in Fig 4, there tended to be more polarization between strict-endorsers and nurturant-endorsers in the metaphor-increase condition, *F*(1, 596) = 54.89, *p* < .001, than in the metaphor-decrease condition, *F*(1, 596) = 16.06, *p* < .001. An examination of the means within each family model yielded a significant effect of condition for the nurturant-endorsers, *F*(1, 596) = 6.87, *p* = .009, *η*_*p*_^*2*^ = .01, but a non-significant difference for the strict-endorsers, *F*(1, 596) = 1.18, *p* = .277, *η*_*p*_^*2*^ < .01.

#### Mediating role of Moral Society attitudes

**Fig 5:**
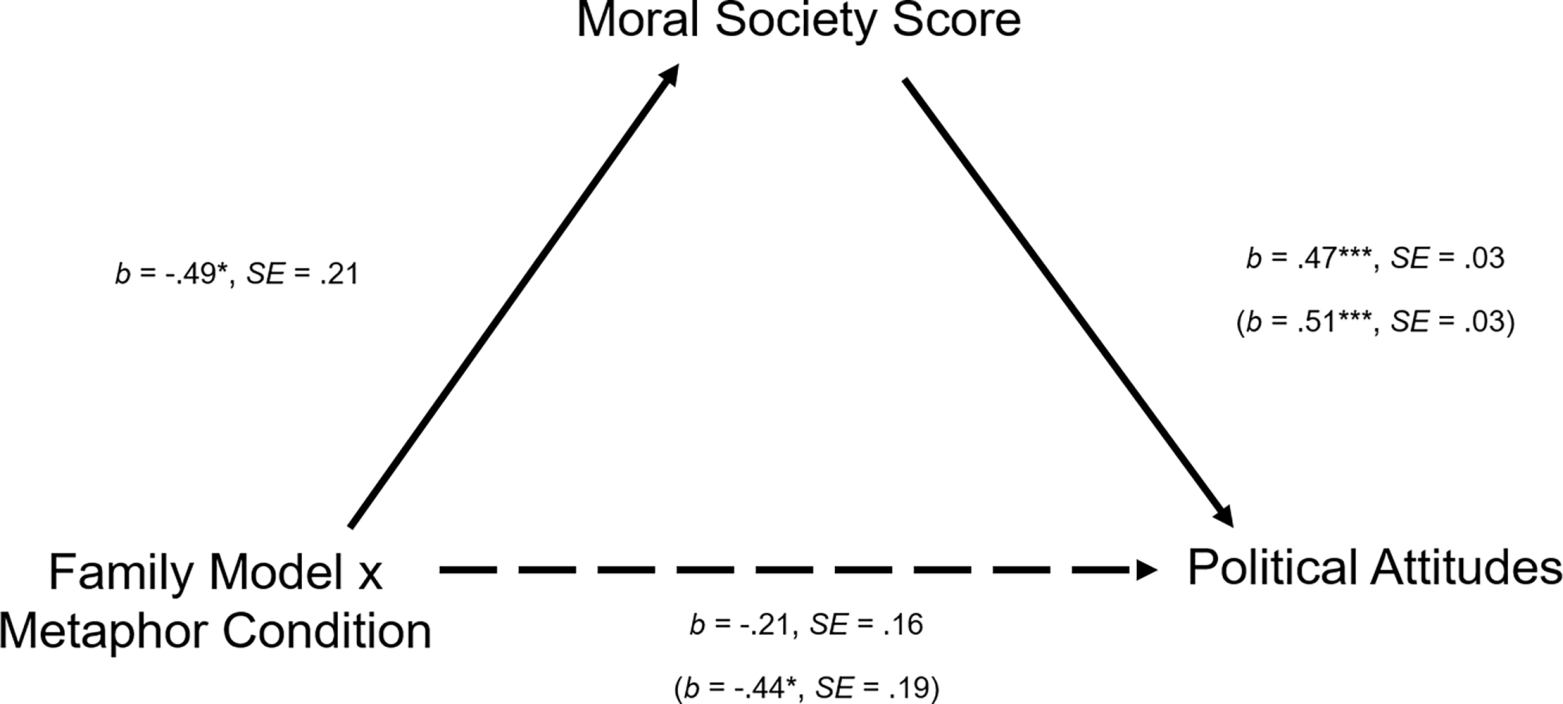
Diagram depicting the mediating role of the Moral Society Scale in explaining the relationship between the Moral Politics Scale x metaphor condition interaction and political attitudes. Standardized regression betas are shown (Study 5). Note: * *p* < .05, ** *p* < .01, and † *p* < .10.

Next, we conducted a mediated moderation analysis [75] to test whether scores on the Moral Society Scale mediated the relationship between parenting beliefs and political attitudes for participants in the metaphor-increase condition. This analysis, using 5000 bootstrap samples, revealed that the 95% confidence interval for the indirect effect did not include 0 [-.42 to -.04] (see Fig 5). This result suggests that participants in the metaphor-increase condition utilized their family ideals as a guide for their political beliefs, which, in turn, guided their political attitudes.

### Discussion–Study 5

In Study 5, we manipulated the extent to which individuals explicitly utilized the nation-as-family metaphor by either encouraging or discouraging its usage. The results showed that participants in the metaphor-increase condition demonstrated a stronger association between parenting beliefs and political attitudes than those in the metaphor-decrease condition. In other words, the relatively greater use of the nation-as-family metaphor led to more political polarization between participants endorsing the strict and nurturant models. Moreover, this relationship was mediated by scores on the Moral Politics Society Scale.

## General discussion

Understanding the nature of political positions–why they cluster together, where they come from–poses a puzzle for the cognitive and social sciences. Moral Politics Theory [1] offers a comprehensive explanation, proposing that conservative and progressive attitudes cluster together as a result of endorsement of one of two distinct moral worldviews, a strict and nurturant worldview. These worldviews are conceptually anchored in people’s beliefs about ideal families, and these family beliefs map onto political attitudes via the nation-as-family metaphor.

MPT has been influential in both academic (e.g., [4, 21, 76, 77]) and political circles (e.g., [2, 3]), yet many of its core assertions to date have lacked empirical validation. While recent studies have shown that the strict and nurturant worldviews are independent and conceptually coherent belief systems that predict endorsement of conservative and progressive stances, respectively [9], until the present research, no research had explored MPT’s assertion that the family models directly map onto political attitudes. Further, MPT’s claim that the nation-as-family metaphor is the nexus between family ideals and political judgment also lacked empirical testing. The purpose of the present research was to fill this gap in the literature.

In line with MPT’s claim that strict and nurturant family models map onto political attitudes, Studies 1 and 2 found that experimentally triggering endorsement of strict and nurturant beliefs in the family domain increased polarization in the political domain. Strict-endorsers led to make arguments in support of their strict parenting beliefs subsequently demonstrated stronger conservative political attitudes than strict-endorsers in a control condition. Likewise, nurturant-endorsers in the manipulation condition tended to subsequently demonstrate stronger progressive attitudes than nurturant-endorsers in a control condition. In contrast, we found that having progressives and conservatives make arguments in support of their political policy positions did not significantly influence subsequent strict or nurturant beliefs in the family domain (Study 3), helping to rule out potential alternative explanations for our findings in Studies 1 and 2. Additionally, Studies 4–5 found both correlational and experimental support for MPT’s argument that the nation-as-family metaphor serves as a conceptual conduit linking individuals’ family beliefs with their social and political attitudes. In Study 4, we found that endorsement of the nation-as-family metaphor moderated the relationship between family beliefs and social and political attitudes. The more participants overtly utilized the metaphor, the stronger the correlation was between their family beliefs and their social and political attitudes. In Study 5, we experimentally manipulated participants’ active endorsement of the nation-as-family metaphor, finding that strict-endorsers and nurturant-endorsers in the metaphor-decrease condition demonstrated significantly less political polarization than strict-endorsers and nurturant-fathers in the metaphor-increase condition. Overall, then, the present research provides a robust step toward empirically validating many of MPT’s key claims.

### Broader implications

Beyond providing an empirical test of many of MPT’s core assertions, the results from the present research advance our larger understanding of the conceptual foundations of political and moral reasoning.

#### Moving beyond single traits

Going beyond other morality research, which mostly describes single traits associated with being either progressive or conservative, the present research not only presents a unique set of moral traits at the foundation of conservatism and progressivism but also explains why such traits and characteristics overlap with one another in larger conceptual constellations. Most moral-political research traces preferences for one or more solitary moral traits as the source of conservatism and progressivism (e.g., [16, 26, 27, 78–82]), such as preferences for fairness, purity, or authority (e.g., [16]). However, while these findings shed valuable light onto aspects of moral cognition as the basis of political reasoning, they treat morality as a bundle of traits that more or less coincidently co-occur in individuals’ reasoning, with no answer to the question of why certain moral beliefs should coalesce in people’s minds. MPT accounts for moral beliefs as the foundation of political judgment in terms of semantically coherent, larger outlooks on the world, whose components are connected in a meaningful way and give rise to each other based on real-world experiences with authority and group membership in family life [1, 9, 34]. As such, MPT and the results we have found in the present research, provide a greater understanding of why various moral traits–both those MPT proposes, and many of those other research has found to predict political ideology–cohere together in particular ways, where their experiential basis lies, and how they hang together in larger cognitive models.

#### Integrating cognitive science with political psychology

The present research also reveals how valuable it can be to integrate cognitive science–the study of how the human conceptual system functions, and how it is connected to our bodies and world experiences–with moral and political psychology and political science research. MPT proposes early family experiences as the experiential basis for making sense of larger, more abstract groups (e.g., the nation), building on cognitive science work on embodiment, which contends that in order to reason about abstract ideas–things one cannot touch, see, smell, or in any other way directly experience–our cognitive apparatus automatically turns to knowledge that is derived from direct interactions with the world (e.g., [28–32]). Additionally, MPT’s assertion that the nation-as-family metaphor facilitates the recruitment of individuals’ early family experiences as a means for reasoning about more abstract social domains builds on well-established cognitive science research on conceptual metaphor (e.g., [33, 36, 47–52, 83]). By extending cognitive science, and especially its findings on embodiment and conceptual metaphor, to moral and political cognition, MPT goes beyond the more typical psychological investigation of political ideology and attitudes, and as a result, offers a more in-depth understanding of the foundations of political cognition. We believe that this integration could yield further discoveries in the future that might not be possible to make by examining moral-political cognition only from the perspective of one discipline.

#### Morality causes politics

While there is a growing literature pointing to a fundamental relationship between morality and political attitudes (e.g., [1, 16–21, 24, 26, 71, 84]), most research shows correlations between morality and politics but does not investigate a possible causal link between moral beliefs and political conservatism and progressivism. The present research is one of the first empirical examinations to show a unidirectional, causal link between morality and politics, where moral judgment in the metaphoric source domain “family” directly impacts moral judgment in the metaphoric target domain “nation”. Results from Studies 1 through 3 demonstrated that the increased endorsement of strict and nurturant morality within the “family” domain directly caused shifts towards more conservative and progressive political outlooks and policy stances, respectively, while the same was not found for the opposite direction. As such, our results suggest that values can play a causal role in the formation and endorsement of political attitudes (c.f., [85, 86]).

#### Strict and nurturant morality across social domains

It is a common assumption in morality research that different domains of social life afford different values. For instance, “religious” and “political” values are often treated as distinct from each other (e.g., [10, 18, 87]). MPT affords a different view on this matter. Namely, the nation-as-family metaphor is a subcase of a more general metaphoric mapping, by which governing institutions–i.e., social groups that include authorities–are construed in terms of the family domain. Thus, individuals’ idealized family models give rise to their general moral worldviews, across social domains. Therefore, the strict and nurturant models should serve as conceptual template for appropriate behavior in many domains of social interaction, such as religious communities, workplaces, schools, and universities [1, 34, 58, 71]. For instance, a prototypical strict-endorser will likely utilize strict beliefs in his or her family life, professional life, and political judgment. Discourse analyses afford initial support for this [1, 7, 71, 88]. For instance, Jensen [88] found that conservatives’ and progressives’ portrayals of God frequently resembled strict and nurturant values, respectively, and Swift [7] found that conservatives tend to see Christianity as in line with strict values, while progressives tend to see it as in line with nurturant values. Overall, this suggests that moral reasoning within various and seemingly unrelated social domains share a common basis, and are therefore more intimately and systematically connected to each other than ideology and social science research has commonly assumed (e.g., [10, 18, 87]).

### Remaining questions

Although we found, across five studies, support for some of MPT’s key claims, the present research opens the door to some larger questions that the present studies may shed some light on, but do not fully answer.

Although the present research finds support for MPT’s claim that idealized family models serve as organizing templates for moral reasoning, and, ultimately, political attitudes, we did not explore how individuals acquire these family models. MPT argues that individuals’ early experiences within their families can be a strong basis for the formation of people’s endorsement of either the strict or nurturant models, and this assessment finds indirect support in family socialization research [78, 89–100]. Even so, an important step for future research would be to conduct longitudinal research examining infants and their family environments and continually tracking them through adulthood.

In addition, as important as the early family experience may be, MPT argues that individuals may also acquire beliefs about ideal families and parenting from other social experiences, such as experiences with other families, schools, sports teams, church, or public discourse and mainstream culture [1, 56]. Thus, while the theory holds that one’s primary family experience serves as the conceptual source domain to reason about larger groups later in life, the question of what an individual sees as an ideal family–a strict or nurturant model–does not have to mirror the parenting practices employed by one’s own parents. Along those lines, MPT argues that biographical and individual factors such as who one befriends or what media one consumes might impact one’s strict or nurturant beliefs. Further, having a child of one’s own may result in changes in an individual’s contestations of ideal family interactions. Individuals that endorse a nurturant family model might, for example, find that their children only respond to being punished, resulting in these individuals becoming more open to strict ideals. Future research might delve into these nuances, exploring what experiences impact people’s endorsement of the strict and nurturant models.

### Conclusion

Altogether, we believe that the present research advances our understanding of the nature of conservative and progressive political positions–why they coalesce in the way they do, and how they are rooted in individuals’ everyday moral reasoning. Specifically, the present investigation provides strong evidence in support of two core assertions of Moral Politics Theory [1]. Namely, the notion that beliefs about ideal families and parenting directly impact political judgment, and that the nation-as-family metaphor serves a mediating role in the mapping of family-level beliefs onto political-level attitudes. While political morality research typically targets individuals’ moral concerns on the abstract, societal level, our findings suggest that there exists a deeper level of moral cognition that stems from direct experiences and interaction with social ingroups and authority in the real world and functions as an implicit conceptual template for reasoning about societal or political morality. On a larger scale, the present research also contributes to our understanding of moral cognition in general, and demonstrates the value that results from merging theoretical and empirical work from the cognitive sciences with related research areas, such as social psychology and political science. Such an overlap, we believe, holds a treasure trove of new perspectives and insights about fundamental moral and political puzzles, such as why people support the political policies they do, how moral societal concerns come about, and what types of discourses shape and possibly change individuals’ deeply held moral beliefs.

## Supporting information

S1 Table Random assignment examination and ANCOVAs, including differences across age and gender (Study 1). (DOCX)

S2 Table Random assignment examination and ANCOVAs, including differences across age and gender (Study 2). (DOCX)

S3 Table Random assignment examination and ANCOVAs, including differences across age and gender (Study 3). (DOCX)

S4 Table Random assignment examination and ANCOVAs, including differences across age and gender (Study 5). (DOCX)

S1 File Gender and the strict and nurturant parent models. (DOCX)

S2 File Participant exclusion. (DOCX)

S3 File Baby crying instructions and stimuli (Study 1). (DOCX)

S4 File Twin sister instructions and stimuli (Study 2). (DOCX)

S5 File Policy argument instructions and stimuli (Study 3). (DOCX)

S6 File ANCOVAs including differences across age and gender (Study 4). (DOCX)

S7 File Metaphor increase and decrease instructions and stimuli (Study 5). (DOCX)

S1 Data Study 1 data set. (SAV)

S2 Data Study 2 data set. (SAV)

S3 Data Study 3 data set. (SAV)

S4 Data Study 4 data set. (SAV)

S5 Data Study 5 data set. (SAV)

S1 Appendix Questionnaires used in Studies 1–5. (DOCX)
