# Supplementary material for: A moral house divided: How idealized family models impact political cognition
Source: PLoS One. 2018 Apr 11;13(4):e0193347. doi: 10.1371/journal.pone.0193347 (PMC5894964; doi:10.1371/journal.pone.0193347)
Supplement: S1 Table — (DOCX) [file pone.0193347.s001.docx]

**S1 Table**

*Study 1*

*Random Assignment Examination and ANCOVAs*

Differences in age across conditions

|  | Means (SD) | t-value | p-value |
| --- | --- | --- | --- |
| Control | 36.42 (12.48) | 1.43 | .153 |
| Manipulated | 38.59 (14.07) |  |  |

Differences in gender across conditions (numbers are counts)

|  | Control | Manipulated |
| --- | --- | --- |
| Male | 75 | 70 |
| Female | 78 | 83 |

Χ^2^(1) = .33, *p* = .567.

2(family model: strict vs. nurturant) x 2(experimental condition: control vs. manipulated) ANCOVAs controlling for Age and Gender

Interaction predicting Role of Government: *F*(1, 298) = 7.374, *p* = .007.

Interaction predicting Welfare and Redistribution: *F*(1, 298) = 10.03, *p* = .002.
