# Supplementary material for: A moral house divided: How idealized family models impact political cognition
Source: PLoS One. 2018 Apr 11;13(4):e0193347. doi: 10.1371/journal.pone.0193347 (PMC5894964; doi:10.1371/journal.pone.0193347)
Supplement: S2 Table — (DOCX) [file pone.0193347.s002.docx]

**S2 Table**

*Study 2*

*Random Assignment Examination and ANCOVAs*

Differences in age across conditions

|  | Means (SD) | t-value | p-value |
| --- | --- | --- | --- |
| Control | 35.90 (12.42) | 0.64 | .520 |
| Manipulated | 36.66 (12.89) |  |  |

Differences in gender across conditions (numbers are counts)

|  | Control | Manipulated |
| --- | --- | --- |
| Male | 123 | 97 |
| Female | 129 | 109 |

Χ^2^(1) = .14, *p* = .714.

2(family model: strict vs. nurturant) x 2(experimental condition: control vs. manipulated) ANCOVAs controlling for Age and Gender

Interaction predicting Role of Government: *F*(1, 445) = 9.79, *p* = .002.

Interaction predicting Welfare and Redistribution: *F*(1, 447) = 7.04, *p* = .005.
