# Supplementary material for: A moral house divided: How idealized family models impact political cognition
Source: PLoS One. 2018 Apr 11;13(4):e0193347. doi: 10.1371/journal.pone.0193347 (PMC5894964; doi:10.1371/journal.pone.0193347)
Supplement: S3 Table — (DOCX) [file pone.0193347.s003.docx]

**S3 Table**

*Study 3*

*Random Assignment Examination and ANCOVAs*

Differences in age across conditions

|  | Means (SD) | t-value | p-value |
| --- | --- | --- | --- |
| Control | 36.48 (12.66) | .48 | .633 |
| Manipulated | 37.05 (12.98) |  |  |

Differences in gender across conditions (numbers are counts)

|  | Control | Manipulated |
| --- | --- | --- |
| Male | 109 | 101 |
| Female | 135 | 111 |

Χ^2^(1) = .40, *p* = .526.

2(family model: strict vs. nurturant) x 2(experimental condition: control vs. manipulated) ANCOVAs controlling for Age and Gender

Interaction predicting Parenting Siblings Beliefs: *F*(1, 444) = 1.94, *p* = .165.

Interaction predicting Strict Parenting Beliefs: *F*(1, 443) = .05, *p* = .822.

Interaction predicting Nurturant Parenting Beliefs: *F*(1, 443) = .69, *p* = .407.
