# Supplementary material for: A moral house divided: How idealized family models impact political cognition
Source: PLoS One. 2018 Apr 11;13(4):e0193347. doi: 10.1371/journal.pone.0193347 (PMC5894964; doi:10.1371/journal.pone.0193347)
Supplement: S4 Table — (DOCX) [file pone.0193347.s004.docx]

**S4 Table**

*Study 5*

*Random Assignment Examination and ANCOVAs*

Differences in age across conditions

|  | Means (SD) | t-value | p-value |
| --- | --- | --- | --- |
| Increase | 36.07 (13.40) | 1.18 | .237 |
| Decrease | 37.37 (13.38) |  |  |

Differences in gender across conditions (numbers are counts)

|  | Increase | Decrease |
| --- | --- | --- |
| Male | 140 | 139 |
| Female | 154 | 166 |

Χ^2^(1) = .25, *p* = .616.

2(family model: strict vs. nurturant) x 2(experimental condition: control vs. manipulated) ANCOVAs controlling for Age and Gender

Interaction predicting Moral Society: *F*(1, 588) = 5.06, *p* = .025.

Interaction predicting Political Attitudes: *F*(1, 588) = 5.91, *p* = .015.
