# Supplementary material for: A moral house divided: How idealized family models impact political cognition
Source: PLoS One. 2018 Apr 11;13(4):e0193347. doi: 10.1371/journal.pone.0193347 (PMC5894964; doi:10.1371/journal.pone.0193347)
Supplement: S1 File — (DOCX) [file pone.0193347.s005.docx]

**S1 File**

*Gender and the strict and nurturant parent models*

While Moral Politics Theory (Lakoff, 1996) labels the two family models it holds to lie at the basis of moral-political reasoning the “strict-father” and “nurturant-parent” models, some scholars use the labels “strict-parent” and “nurturant-parent” to acknowledge that strictness and the disciplining of children is not an essentially masculine trait (Haas, Fischman, and Brewer, 2015). This is in line with a vital argument in original Moral Politics Theory (1996), namely the fact that the strict and nurturant models constitute what cognitive science calls idealized cognitive models, i.e., simplified representations of the world that govern individuals’ everyday reasoning about the world but must not always match reality and very commonly derive from reality (e.g., Lakoff, 1987a, 1987b; Croft & Cruse, 2004, p. 28). Since the strict and nurturant models have been posited as idealized cognitive models (Lakoff, 1996), MPT holds it entirely possible – and, in actuality, sees it as quite common – that women act as metaphoric “strict fathers” in family life and that, likewise, men are not in essence less likely to engage in nurturant parenting than women. The reason, then, to assign the “strict father”-label to the strict model lies in the fact that central parts of this model do evolve around the notion of male authority. For instance, men are seen in this idealized cognitive model as holding natural authority over women and as being more capable to defend a family against evil as well as implement punishment within the family where necessary. The nurturant-parent model, in contrast, does not assume gendered authority, partly because there is no comparable concern with one absolute authority in the family and physical strength as it relates to defending one’s ingroup against evil and disciplining children as well as, if necessary, physically force them to follow the strict rules set forth by the parental authority. In conclusion, while the “strict-father” label aligns with the model’s central concern with strong, male authorities and a natural order that sets men above women, the label commonly seems to infer that men are more prone to endorse the strict model, while women are more prone to endorse the nurturant model.
