# Supplementary material for: A moral house divided: How idealized family models impact political cognition
Source: PLoS One. 2018 Apr 11;13(4):e0193347. doi: 10.1371/journal.pone.0193347 (PMC5894964; doi:10.1371/journal.pone.0193347)
Supplement: S2 File — (DOCX) [file pone.0193347.s006.docx]

**S2 File**

*Participant exclusion*

It is possible that by removing participants who reported being strict-father or nurturant-parent but subsequently selected the contrasting position in the parenting scenario we may have biased the results. If these removed participants endorse both strict-father and nurturant-parent ideals, they might be biconceptuals, and therefore may tend toward more moderate political positions. Thus, removing these individuals from the manipulated condition might have created a selection bias where fewer moderates were in the manipulated condition than the control condition which could explain why strict-fathers and nurturant-parents in the manipulated condition reported more extreme political stances.

However, if such a biased sample existed in the manipulated condition, participants’ responses in that condition should have a larger standard deviation than responses in the control condition since participants in the manipulated condition would be more polarized (before the manipulation). Yet, we found the following standard deviations: Study 1, *Role of Government*: SF_control_= 1.11, SF_manipulated_= 1.11; NP_control_= 1.00, NP_manipulated_= 0.98; *Welfare and Redistribution Attitudes*: SF_control_= 0.86, SF_manipulated_= 0.79; NP_control_= 0.78, NP_manipulated_= 0.74. Study 2, *Role of Government*: SF_control_= 1.03, SF_manipulated_= 1.14; NP_control_= 1.09, NP_manipulated_= 0.90; *Welfare Attitudes*: SF_control_= 0.92, SF_manipulated_= 1.01; NP_control_= 1.01, NP_manipulated_= 0.77; *Social Justice*: SF_control_= 0.70, SF_manipulated_= 0.82; NP_control_= 0.90, NP_manipulated_= 0.80. Levine’s Test of equality of variances yielded no significant difference between variances for any of the dependent measure for strict-fathers in the control and strict-fathers in the manipulated conditions, *F*s < 2.44, *p*s > .12. The test yielded a significant difference in variances between nurturant-parents in the control and nurturant-parents in the manipulated condition in two of the five dependent variables (Study 2’s *Role of Government*, *F* = 5.69, *p* = .018, and *Welfare Attitudes*, *F* = 4.67, *p* = .032). However, there was no consistent pattern for which condition’s variance was larger: *Role of Government* variance for the manipulated condition was higher than the variance in the control, whereas the pattern was reversed for *Welfare Attitudes*.

Additionally, if our results were due to a selection bias that caused moderate participants to be removed from the manipulated condition, then the difference between the “removed” and “kept” participants would show up in scores on the political ideology scale measured in Study 2 (i.e., kept strict-fathers would score higher on conservatism than removed strict-fathers, and kept nurturant-parents would score higher on liberalism than removed nurturant-parents). A comparison between the removed and kept strict-fathers yielded no significant difference in political ideology scores, *t*(188) = -.783, *p* = .435. Likewise, there was no significant difference in political ideology for the removed and kept nurturant-parents, *t*(371) = .148, *p* = .882. Altogether, these analyses suggest that the results found in both Studies 1 and 2 were not due to a selection bias.

*Analyses with All Participants (including Biconceptuals)*

If we include the participants who indicated either being strict or nurturant but then went on to select the parenting option that fit with the other family model in the analyses (i.e., the biconceptuals), we find the following results:

*Study 1*:

The 2(family model: strict vs. nurturant) x 2(experimental condition: control vs. manipulated) ANOVA predicting Role of Government yielded a significant interaction *F*(1, 401) = 4.46, *p* = .035, indicating greater polarization between strict-endorsers and nurturant-endorsers when in the manipulated condition. Specifically, the strict-endorsers tended to score lower in the manipulated condition, *M* = 2.84, than the strict-endorsers in the control condition, *M* = 3.13, *F*(1, 401) = 2.44, *p* = .119, while the nurturant-endorsers tended to score higher in the manipulated condition, *M* = 3.81, than the nurturant-endorsers in the control condition, *M* = 3.61, *F*(1, 401) = 2.05, *p* = .153.

The 2(family model: strict vs. nurturant) x 2(experimental condition: control vs. manipulated) ANOVA interaction predicting Welfare and Redistribution yielded a marginal result, *F*(1, 401) = 3.54, *p* = .061, suggesting a trend toward more polarization between the strict-endorsers and nurturant-endorsers when in the manipulated condition. Simple comparisons indicated that strict-endorsers in the manipulated condition tended to score lower, *M* = 2.97, than strict-endorsers in the control condition, *M* = 3.14, *F*(1, 401) = 1.59, *p* = .208, while nurturant-endorsers in the manipulated condition condition tended to score higher, *M* = 3.75, than nurturant-endorsers in the control condition, *M* = 3.60, *F*(1, 401) = 2.10, *p* = .148.

The results suggest that, even though the the inclusion of these biconceptual participants increased the sample size and therefore statistical power, their inclusion weakened our results. This suggests that the manipulation affected non-biconceptuals more than the biconceptuals. This may be due to the fact that when participants in the manipulated condition were asked to choose which of the two parenting options they believed to be the correct option, they were presented with one strict and one nurturant option. Since biconceptuals are susceptible to influence from arguments framed either as strict or nurturant (see Wehling et al. 2017), the presentation of both options at the same time likely minimized the influence that either option had on the biconceptuals (i.e., the frames cancelled each other out).

*Study 2:*

The 2(family model: strict vs. nurturant) x 2(experimental condition: control vs. manipulated) ANOVA predicting Role of Government yielded a significant interaction *F*(1, 560) = 4.78, *p* = .029, indicating that there was greater polarization between strict-endorsers and nurturant-endorsers when in the manipulated condition. There was a marginally significant trend for strict-endorsers to score lower when in the manipulated condition, *M* = 3.06, than when in the control condition, *M* = 3.34, *F*(1, 560) = 3.43, *p* = .064, whereas the nurturant-endorsers tended to score higher in the manipulated condition, *M* = 3.74, than the nurturant-endorsers in the control condition, *M* = 3.62, *F*(1, 560) = 1.36, *p* = .243.

The 2(family model: strict vs. nurturant) x 2(experimental condition: control vs. manipulated) ANOVA predicting Welfare and Redistribution yielded a significant interaction, *F*(1, 562) = 4.04, *p* = .045, suggesting more polarization between the strict-endorsers and nurturant-endorsers when in the manipulated condition. Simple comparisons indicated that strict-endorsers in the manipulated condition tended to score lower, *M* = 3.08, than strict-endorsers in the control condition, *M* = 3.34, *F*(1, 562) = 3.64, *p* = .057, while nurturant-endorsers in the manipulated condition condition tended to score higher, *M* = 3.74, than nurturant-endorsers in the control condition, *M* = 3.66, *F*(1, 562) = .62, *p* = .430.

The 2(family model: strict vs. nurturant) x 2(experimental condition: control vs. manipulated) ANOVA interaction predicting Social Justice yielded a marginal result, *F*(1, 568) = 3.10, *p* = .079, suggesting a trend toward more polarization between the strict-endorsers and nurturant-endorsers when in the manipulated condition. Simple comparisons indicated that strict-endorsers in the manipulated condition tended to score lower, *M* = 2.84, than strict-endorsers in the control condition, *M* = 3.08, *F*(1, 568) = 4.14, *p* = .042, while nurturant-endorsers in the manipulated condition condition tended to score higher, *M* = 3.42, than nurturant-endorsers in the control condition, *M* = 3.40, *F*(1, 568) = .03, *p* = .862.

See section above in Study 1 for discussion of why including the biconceptuals likely weakened the overall results.
