# Supplementary material for: A moral house divided: How idealized family models impact political cognition
Source: PLoS One. 2018 Apr 11;13(4):e0193347. doi: 10.1371/journal.pone.0193347 (PMC5894964; doi:10.1371/journal.pone.0193347)
Supplement: S3 File — (DOCX) [file pone.0193347.s007.docx]

**S3 File**

Study 1

*Baby Crying Instructions and Stimuli*

Control Condition

“Now, think about an average day in your life. What is your normal routine? Please spend a few minutes describing your normal routine below (write at least 5 sentences). Be as clear as possible.” Participants were then presented with a text box where they could describe their average day.

Manipulated Condition

“It is nighttime and your baby is crying in his bed for no apparent reason. You already checked to make sure he is not sick, hungry, or uncomfortable. Even though all is fine he keeps crying loudly and this has been going on for some time. What do you do as a parent?”

Parenting Options

*Nurturant Model*:

Parent A:

When it comes to parenting, I believe in being nurturing. When my baby cries at night for no apparent reason, I pick him up. I soothe him until he calms down. The baby is seeking parental attention and simply is uncomfortable being by himself. In this situation, the most important thing for the baby is to be held, soothed, and comforted by his parents as to feel that he is not left alone in this world. Leaving the baby to himself until he exhausts himself from crying is wrong, because it will make the child feel like he is not nurtured and cared for. An approach that is based on empathy, unconditional love and nurturance for the child is the best parenting solution here.

*Strict Model*:

Parent B:

When it comes to parenting, I believe in being strict. When my baby cries at night for no apparent reason, I do not pick him up. I let him cry until he calms himself down. The baby is seeking parental attention and simply is uncomfortable being by himself. In this situation, the most important thing for the baby is to be left alone and learn to soothe himself without relying on his parents. Parents that rush to comfort their babies every time they cry do not do them a favor at all, because they do not give the child the opportunity to learn how to cope with distress on his own and become self-dependent. An approach that avoids excessive parental indulgence of the child and instead gives him a chance to find ways to rely on himself is the best parenting solution here.
