# Supplementary material for: A moral house divided: How idealized family models impact political cognition
Source: PLoS One. 2018 Apr 11;13(4):e0193347. doi: 10.1371/journal.pone.0193347 (PMC5894964; doi:10.1371/journal.pone.0193347)
Supplement: S4 File — (DOCX) [file pone.0193347.s008.docx]

**S4 File**

Study 2

*Twin Sister Instructions and Stimuli*

You have twin girls who are four years old. Both spent the afternoon raking leaves in the neighbor’s yard to make some money. The neighbor pays them by square feet. Both kids work hard all afternoon. One is very concentrated and organized and she gets a lot of square feet covered. The other has a slight attention deficit and gets easily distracted – although she worked equally hard all day she didn’t get as many square feet covered. As a result, she only gets half of the money her sister made. She is sad and disappointed to have worked so hard but not succeeded as much as her sister. What do you do as a parent?

*Nurturant Parent*:

Parent A:

Children should get what they need and wish for, and not simply what they “earned for themselves”. Therefore, I would give the child that was less successful money to make up for the difference. I wouldn’t give the other one any extra money, because she did well for herself. This way both kids are looking at a fair, equal outcome of their efforts. The only way to treat siblings right is to give them what they each need, even if that means giving them different things. Parents who fail to individually support children are wrong, because they don’t give each sibling what is needed to lead a happy and self-fulfilled life. An approach that is based on individual empowerment and need-based support is the best parenting solution here.

*Strict Father*:

Parent B:

Children should get what they earn for themselves, this is the only way they will understand that life works this way – you get what you deserve and what you earn for yourself! Therefore, I would not give the child that was less successful money to make up for the difference. She needs to get motivated to do better and discipline herself to focus, and she will not learn this by being given something she does not deserve. That type of immoral indulgence will only make her weak and she won’t learn to be better. It’s also unfair to her sister, who needs to see that working hard and getting better results is rewarded accordingly. Parents who fail to teach children that people get what they earn for themselves are wrong. An approach that doesn’t mess with the natural reward system in life will teach the kids to try harder and it the best parenting solution here.
