# Supplementary material for: A moral house divided: How idealized family models impact political cognition
Source: PLoS One. 2018 Apr 11;13(4):e0193347. doi: 10.1371/journal.pone.0193347 (PMC5894964; doi:10.1371/journal.pone.0193347)
Supplement: S5 File — (DOCX) [file pone.0193347.s009.docx]

**S5 File**

Study 3

*Policy Argument Instructions and Stimuli*

Many Americans work hard to make ends meet. Some come from backgrounds that make it easy for them to succeed in life. Others come from backgrounds with little wealth, little education, or face issues because of their sex, race, or religion. Despite the fact that these people work as hard as they can, financial and social success is harder to come by for them, and some doors seem to be closed to them altogether. What, if anything, should the government do?

*Progressive*:

Argument A

The government should give extra assistance to those in need of extra support. People whose background does not easily grant them access to good chances in life – good education, successful careers, and employment – should be supported as much as needed by public assistance programs. It would be wrong not to assist them. People should receive assistance from the government in accordance to their individual needs.

*Conservative*:

Argument B

The government should treat everyone equally and provide all citizens with the same basic level of support and protection, no matter their individual background. Giving those who have had less success extra attention, help, or money doesn't help them succeed. Rather, it is more important to let the market dictate what compensation they get for their efforts. The government should treat all people equally. No one should expect special treatment simply because of their background.
