# Supplementary material for: A moral house divided: How idealized family models impact political cognition
Source: PLoS One. 2018 Apr 11;13(4):e0193347. doi: 10.1371/journal.pone.0193347 (PMC5894964; doi:10.1371/journal.pone.0193347)
Supplement: S6 File — (DOCX) [file pone.0193347.s010.docx]

**S6 File**

Study 4

*Analyses controlling for gender and age*

Interaction between Moral Politics Scale and Nation-as-Family Metaphor Engagement predicting political attitudes, controlling for age and gender: *b* = .07, *S.E.* = .04, *p* = .035.

Interaction between Moral Politics Scale and Nation-as-Family Metaphor Engagement predicting Moral Society scores, controlling for age and gender: *b* = .07, *S.E.* = .03, *p* = .046.
