# Supplementary material for: A moral house divided: How idealized family models impact political cognition
Source: PLoS One. 2018 Apr 11;13(4):e0193347. doi: 10.1371/journal.pone.0193347 (PMC5894964; doi:10.1371/journal.pone.0193347)
Supplement: S7 File — (DOCX) [file pone.0193347.s011.docx]

**S7 File**

Study 5

*Metaphor Increase and Decrease Instructions and Stimuli*

Metaphor-increase condition

There’s a very common metaphor that people use when reasoning and talking about national politics, a metaphor used by conservatives, progressives, and moderates alike. It goes like this: ‘running a nation is in many ways just like running a family.’ In many ways, this metaphor seems to be applicable. For example, one might say that just as parents have authority and lead the family so do government officials have authority and lead the nation. Nations are groups of people and families are groups of people. And so on. We are curious whether you can think of more examples that show how well this metaphor works - please think of some other ways in which the nation is JUST like a family. Simply list 4-5 examples.

Metaphor-decrease condition

There’s a rather silly metaphor that people use when reasoning and talking about national politics, a metaphor used by conservatives, progressives, and moderates alike. It goes like this: ‘running a nation is in many ways just like running a family.’ This metaphor is inadequate in many ways. For example, while people in a family are related it isn't the case that all people in one nation are related. And while you can chose to move to a different nation you cannot chose to move into a different family in the same way. And so on. We are curious whether you can think of more examples that show how inadequate this metaphor is - please think of some other ways in which the nation is NOT like a family. Simply list 4-5 examples.
