# Supplementary material for: A moral house divided: How idealized family models impact political cognition
Source: PLoS One. 2018 Apr 11;13(4):e0193347. doi: 10.1371/journal.pone.0193347 (PMC5894964; doi:10.1371/journal.pone.0193347)
Supplement: S1 Appendix — (DOCX) [file pone.0193347.s017.docx]

**S1 Appendix**

**Welfare and Redistribution Attitudes (Studies 1 & 2)**

It’s important we have a solid, well-functioning welfare system.

It’s a government’s job to provide citizens with basic things they need to live happy, fulfilled lives.

Welfare is generally a good idea.

Government programs that help the socially disadvantaged should be expanded.

Government should never leave citizens without medical care, food, or shelter.

People are poor due to circumstances beyond their control.*

I would support a program that resulted in higher taxes to support social programs for poor people.*
People who are poor should not be blamed for their misfortune.*

Society has the responsibility to help poor people.*

*Only used in Study 1

**Social Justice Political Attitudes Scale (Study 2)**

It’s not the government’s responsibility to reduce income inequality between the rich and poor.

Real equality means people get to keep what they earn in the free market.

Redistribution of income is unfair to those who work hard.

Government programs must support people of different backgrounds based on their individual needs.

We should put more tax money towards empowering people via a good, public education system.

**Political Ideology (Study 2 & 4)**

Please indicate how politically liberal or conservative you consider yourself to be on social issues.

Please indicate how politically liberal or conservative you consider yourself to be on economic issues.

How much do you tend to like or dislike political conservatives?*

How much do you tend to like or dislike political liberals?*

How warm or cold do you feel toward each of the following.  Please use this “thermometer” to indicate your feelings toward each.  A “0” indicates that you have very cold feelings, whereas a “100” indicates that you have very warm feelings. *—Liberals –Conservatives*

*Only used in Study 2

**Parenting Siblings Scale (Study 3)**

Strong competition between siblings is healthy

It’s good for children to learn early that failure is a signal that one must work harder.

Siblings should compete with each other.

Parents should support their children based on each child’s individual needs.

Even if one sibling is doing worse than the other, parents should not provide extra help to the child who is failing. Rather, that child needs to become self-sufficient and learn how succeed on his or her own.

**Moral Politics Scale (Study 3 & 4)**

*Strict-father subscale*

While other people must not be one’s concern, within a family, everyone should look after each other. (

Obedience must be instilled in children.

When grownups talk children ought to be quiet.

Parents shouldn’t handicap their children by making their lives too easy.

Bad behavior in children must be punished sufficiently.

I will not have my child talk back to me.

Children must always be on time.

“Tough love” is required to raise a child right.

Children need to be disciplined in order to build character.

Children must be taught that people get what they deserve.

Children must be disciplined through strict rules at home.

It’s fine for children to have secrets and hide things from their parents.

When in doubt, parents should err on the side of lenience rather than strictness.

At times it’s okay for children to disobey their parents.

Sometimes it’s okay to let bad behavior in children go unpunished.

*Nurturant-parent subscale*

Children will grow up to be happy adults if parents encourage them to follow their curiosity.

Children should learn to understand others’ needs and attend to them.

Children must learn to see the world through other people’s eyes.

I would rather see my child play cooperatively than play competitively.

In order to truly nurture children one needs to be empathic.

Parenting means nurturing the child’s true nature.

Siblings should receive parental support in accordance to their individual needs.

Parents should empower children as much as possible so that they may follow their dreams.

Knowing how to care for others is not a central thing for a child to learn.

Children shouldn’t feel obligated to care about the well-being of people they do not know. Tending to the needs of others is not a sign of responsibility in children.

It’s not critical for children to learn to take the perspective of others into account.

Learning to understand others and accepting them for who they are is not important for children to learn.

It’s not important for parents to explain to their children why they set certain rules and limits.

**Nation-as-Family Metaphor Engagement Questionnaire (Study 4)**

Although the citizens of one nation are not all related to each other, they are in a way “part of one big family.”

Parents and the government share similar responsibilities – while parents look out for their children, government looks out for its citizens.

Just like parents care about their children, the government cares about its citizens.

Nations and families have little in common.

Nations are nothing like families.

**Moral Society Scale (Study 4 & 5)**

*Strict-father subscale*

While citizens of other nations must not be one's concern, within the US, everyone should look after each other.

The government must instill obedience in its citizens.

When the government speaks, Americans ought to listen respectfully.

The government shouldn't handicap its citizens by making their lives too easy.

Unlawful behavior must be punished sufficiently.

Government authorities should not allow citizens to talk back to them.

People must always be on time.

Sometimes the government needs to practice "tough love” to ensure its citizens follow the right path.

Citizens need to be disciplined in order to build character.

People must understand that people get what they deserve.

Citizens must be disciplined through strict rules and laws.

It's fine for citizens to have secret dealings and hide things from the government.

When in doubt, the government should err on the side of lenience rather than strictness.

At times it's okay for citizens to disobey the government's laws.

Sometimes it’s okay to let bad behavior in citizens go unpunished.

*Nurturant-parent subscale*

Citizens will be happy if the government encourages them to follow their curiosity.

People in America should learn to understand others' needs and attend to them.

Citizens must learn to see the world through other people's eyes.

I'd rather see America work cooperatively with other nations than be in competition with them.

In order to truly take care of its citizens, the government needs to be empathic.

Governing means nurturing the true nature of each citizen.

Americans should receive governmental assistance in accordance to their individual needs.

The government should empower its people as much as possible so that they may follow their dreams.

Caring for others is not a central aspect of being American.

Americans shouldn’t feel obligated to care about the well-being of citizens from other nations.

Tending to the needs of those in other nations is not the responsibility of Americans.

It’s not critical for people to learn to take the perspective of others into account.

Learning to understand others and accepting them for who they are is not an important part of being American.

It’s not important for the government to explain to its people why it set certain rules and laws.

**Hot-Button Political Attitudes Scale (Study 5)**

TAXES: Increase income taxes for wealthier Americans (couples making at least $250,000 together, or individuals making $200,000).

HEALTH CARE: Overturn the federal legislation mandating that all Americans have health insurance.

ENVIRONMENTAL POLICY: Pass federal legislation that taxes corporations for emitting carbon dioxide into the air (a carbon tax).

GAY MARRIAGE: Pass federal legislation making marriage between same-sex individuals legal.

IRAN: Use military force (e.g., airstrikes) to force Iran to terminate its nuclear weapons program.

ABORTION: Make having an abortion illegal (overturning Roe vs. Wade).

IMMIGRATION: Deport back to their home country all illegal immigrants living within the United States.

WELFARE: Stop paying welfare to those who do not find a job after 6 months.

SYRIAN CIVIL WAR: Implement a "no-fly" zone over Syria so no Syrian planes can fly.

MILITARY SPENDING: Increase the amount of money spent on the military.
